# Magnetic 2D Transition-Metal-Based Nanomaterials in Biomedicine: Opportunities and Challenges in Cancer Therapy

**DOI:** 10.3390/ma18112570

**Published:** 2025-05-30

**Authors:** Sunčica Sukur, Václav Ranc

**Affiliations:** 1 Institute of Molecular and Translational Medicine, Faculty of Medicine and Dentistry, Palacký University and University Hospital, 779 00 Olomouc, Czech Republic; 2 Regional Centre of Advanced Technology and Materials, Czech Advanced Technology and Research Institute, Palacký University Olomouc, 775 15 Olomouc, Czech Republic

**Keywords:** transition metal dichalcogenides, transition metal carbides/nitrides, transition metal oxides, metal–organic frameworks, magnetic nanoparticles, targeted drug delivery, photothermal therapy, photodynamic therapy, hyperthermia, magnetic resonance imaging

## Abstract

Severe systemic toxicity and poor targeting efficiency remain major limitations of traditional chemotherapy, emphasising the need for smarter drug delivery systems. Magnetic 2D transition-metal-based nanomaterials offer a promising approach, as they can be designed to combine high drug loading, precise targeting, and controlled release. The key material classes—transition metal dichalcogenides, transition metal carbides/nitrides, transition metal oxides, and metal–organic frameworks—share important physicochemical properties. These include high surface-to-volume ratios, tuneable functionalities, and efficient intracellular uptake. Incorporating magnetic nanoparticles into these 2D structures broadens their potential beyond drug delivery, through enabling multimodal therapeutic strategies such as hyperthermia induction, real-time imaging, and photothermal or photodynamic therapy. This review outlines the potential of magnetic 2D transition-metal-based nanomaterials for biomedical applications by evaluating their therapeutic performance and biological response. In parallel, it offers a critical analysis of how differences in physicochemical properties influence their potential for specific cancer treatment applications, highlighting the most promising uses of each in bionanomedicine.

## 1. Introduction

Traditional chemotherapy remains a delicate balance between efficacy and toxicity—while it provides over 70 different drugs for cancer treatment, its systemic effects often lead to severe side effects, including blood disorders and nervous system damage [1,2,3]. Cancer persists as a major global health threat and causes a high number of deaths annually [4]. A key challenge in improving the outcomes of cancer therapy lies in minimising unintended harm to healthy tissues by improving targeting and controlled drug release, which is one of the greatest downsides of the treatment at the moment [5,6]. An approach that could make this difference is the development of smart drug delivery systems—systems designed to selectively target cancer cells and release drugs in response to specific stimuli [7,8]. Besides the acidic pH of the tumour microenvironment, which promotes drug release [9], magnetically responsive systems have gained particular attention due to their precision and efficiency in guiding therapeutic agents directly to tumour sites [10].

Nanomaterials have emerged as an irreplaceable component in new smart biomedical systems [11]. Some of their beneficial properties are high surface-to-volume ratio, enhanced reactivity, and the ability to manipulate their size and surface. Among them, magnetite (Fe_3_O_4_) nanoparticles (NPs) stand out due to their biocompatibility, biodegradability, low cost, and strong magnetism [12]. These properties make them highly suitable—alone or as part of a composite—for a range of biomedical applications including magnetic resonance imaging (MRI) [13], targeted drug delivery [14], and photothermal therapy (PTT) [15]. Multifunctional nanocomplexes that incorporate magnetic NPs have the potential to overcome numerous limitations of the classic therapy [16,17].

**Figure 1:**
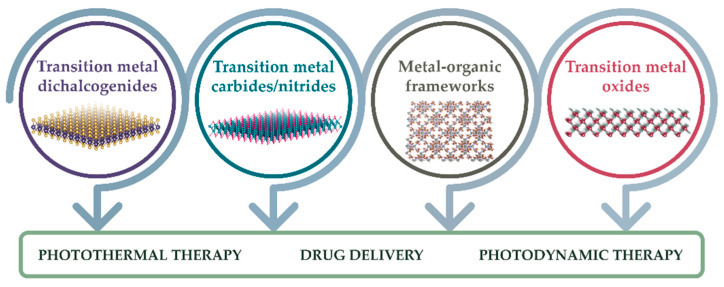
Schematic overview of the four key classes of transition-metal-based 2D nanomaterials—TMDs, MXenes, MOFs, and TMOs—highlighting their main biomedical applications.

Recent research has been focused on two-dimensional (2D) nanomaterials, a rapidly expanding class of nanomaterials known for their high specific surface area and diverse electronic, optical, and catalytic properties [18]. Among their most significant properties as potential theranostic systems are their high surface-to-volume ratios and ability to accumulate in tumours [19,20]. In addition, intracellular uptake evaluations have shown that 2D forms can be taken up more efficiently than other geometries [19]. This enhanced internalisation is largely attributed to their ultrathin, planar morphology, which enables stronger interactions with the cell membrane and facilitates endocytosis. Furthermore, this geometry promotes improved interaction with biological interfaces, enhancing biodistribution and retention within tumour tissue. Beyond structural advantages, the high surface-to-volume ratio of 2D nanomaterials supports greater drug-loading capacity and versatile surface modification, enabling the integration of tailored therapeutic and imaging functionalities within a single platform. This broad category includes various planar, few-nanometre-thick materials with tuneable properties, making them highly suitable for biomedical applications. In particular, this review focuses on transition metal (TM)-based 2D nanomaterials (Figure 1). These include transition metal dichalcogenides (TMDs) [21], transition metal carbides/nitrides (MXenes) [22], metal–organic frameworks (MOFs) [23], transition-metal oxides (TMOs) [24], and certain layered double hydroxides (LDHs) [25]. Chemical versatility and tuneable properties make transition-metal-based 2D nanomaterials strong candidates for cancer therapy; however, it should be stated that systematic biocompatibility evaluation remains the main challenge, although the interest in overcoming it has rapidly increased in the last decade [26].

Transition metals, such as iron (Fe), manganese (Mn), copper (Cu), nickel (Ni), titanium (Ti), and zinc (Zn), are key elements in these materials [27]. They are characterised by high density, strong metallic bonding, and high melting and boiling points that arise from delocalised d-electrons and contribute to cohesion and stability [28]. Many nanomaterials based on these elements possess magnetic properties, and their performance as magnetically guided agents in bionanomedicine could potentially be enhanced by incorporating magnetic nanoparticles, which have been extensively studied for their responsiveness to external magnetic fields [29].

**Figure 2:**
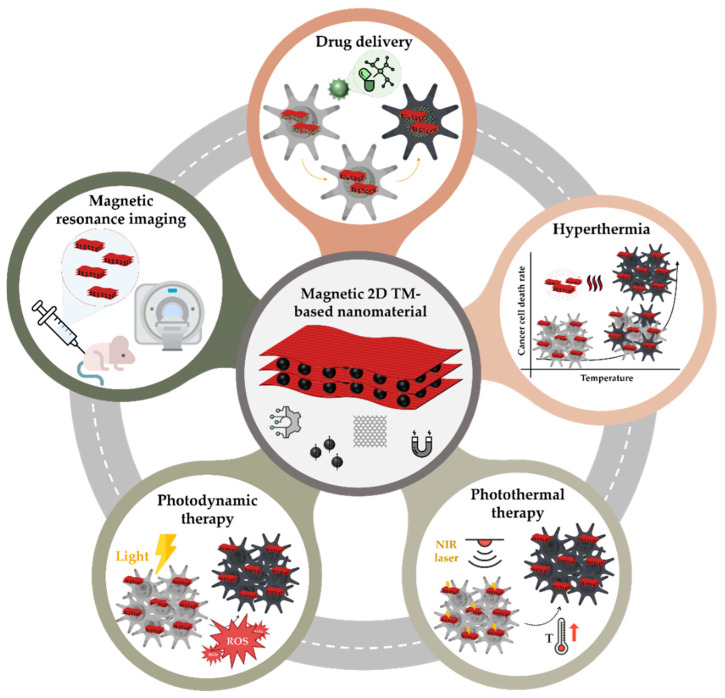
Schematics illustrating the potential applications of magnetic transition-metal-based 2D nanomaterials in cancer therapy discussed in this article.

Considering the important role of magnetism in the development of smart systems for combined biomedical applications [30], along with the chemical versatility, potential for controlled drug release, and high drug-loading capacity of 2D TM-based nanomaterials, combining these materials represents one of the most promising directions for future advancement in the field. Such nanocomposites could be activated under a magnetic field, triggering the release of anticancer drugs while also generating localised hyperthermia through near-infrared (NIR) irradiation [31,32,33,34]. The ultimate goal is to develop a system that ensures targeted, on-demand drug release; minimises toxicity to healthy cells; and follows safe degradation and excretion pathways [35]. Figure 2 illustrates a schematic of the main potential applications of magnetic transition-metal-based 2D nanomaterials in cancer therapy, including drug delivery, hyperthermia, photothermal therapy, photodynamic therapy (PDT), and magnetic resonance imaging.

Given the significant progress in this field, this review aims to integrate current advancements, critically analyse and compare possibilities, and highlight both the strengths and challenges of four key classes of transition-metal-based 2D nanomaterials—TMDs, MXenes, MOFs, and TMOs—with a particular focus on their magnetic composites, which represent some of the most promising platforms for advancing multimodal strategies in nanooncology.

## 2. Transition Metal Dichalcogenides

**Figure 3:**
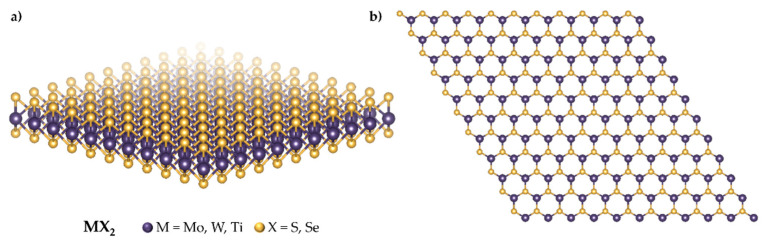
Structure of transition metal dichalcogenides: (**a**) TMDs monolayer; (**b**) top view of TMDs’ structure with trigonal prismatic coordination.

Transition metal dichalcogenides (TMDs) are promising 2D materials for multimodal systems for biomedical applications due to their large surface area and biocompatibility [36,37]. The general formula of TMDs can be expressed as MX_2_, in which M is a transition metal from group 4 to 10 covalently bonded between two hexagonal layers of chalcogen atoms, often in a trigonal prismatic geometry [21] (Figure 3). Typical transition metal dichalcogenides, such as molybdenum disulfide (MoS_2_), tungsten disulfide (WS_2_), titanium disulfide (TiS_2_), molybdenum diselenide (MoSe_2_), and tungsten diselenide (WSe_2_), exhibit a planar crystal structure with unique chemical and optical properties [38]. Compared to graphene, TMDs are more robust, exhibit higher band gaps, and can be tuned using various surface functionalisation techniques [21,39]. There are various ways to synthesise these materials, from mechanical cleavage to chemical intercalation and chemical vapour deposition [40].

TMDs’ structure and properties can support sustainable drug release, NIR photothermal/photodynamic therapy, enzyme immobilisation, 3D printing scaffolds, and tissue engineering [41]. They have shown promise in tumour immunotherapy by modulating the tumour immune microenvironment and enhancing immune cell activity. Their unique physicochemical properties allow for the delivery of immunotherapeutic agents and combination with other treatment modalities, thereby amplifying antitumour immune responses [42]. The enhanced permeability and retention (EPR) effect, central to targeted cancer therapy for decades, facilitates the passive accumulation of TMDs in tumours [43].

Considering their magnetic properties, two-dimensional transition metal dichalcogenides exhibit strong magnetic and magneto-optical behaviours. However, these features are predominantly explored in non-biomedical fields such as spintronics, valleytronics, and quantum information technologies [44,45,46]. As for magnetic phenomena in TMDs for biomedical applications, research still relies on the incorporation of magnetic nanoparticles [47]. Such nanoplatforms are particularly interesting for photothermal therapy and magnetically targeted drug delivery [21,48,49], which is the primary focus of this section.

**Figure 4:**
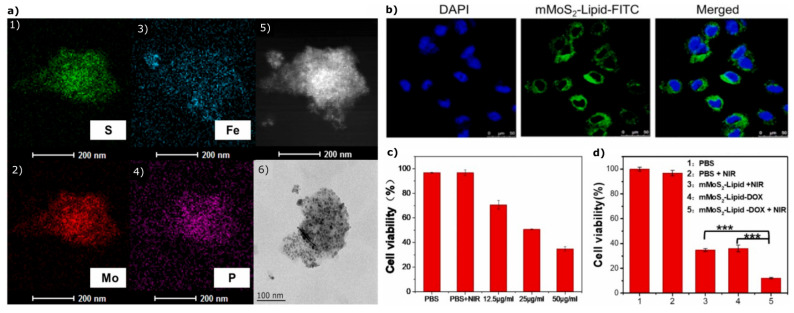
(**a**) TEM elemental mapping images displaying the distribution of sulphur (**1**), molybdenum (**2**), iron (**3**), and phosphorus (**4**), along with TEM micrographs of mMoS_2_-lipid (**5**) and mMoS_2_ (**6**). (**b**) Cellular uptake of FITC-labelled mMoS_2_-lipid (DAPI-labelled cell nuclei). (**c**) Cell viability following treatment with mMoS_2_-lipid at concentrations of 12.5, 25, and 50 μg mL^−1^ after 10 min NIR laser irradiation (2 Wcm^−2^) and incubation for 24 h. (**d**) Cell viability assessment after 24 h incubation (PBS, PBS + NIR, mMoS_2_-lipid + NIR, mMoS_2_-lipid-DOX, mMoS_2_-lipid-DOX + NIR) incubating with cells for 24 h at 50 μg mL^−1^ concentration of doxorubicin (mean ± SD, *n* = 3; *** statistical significance). Reproduced from [1], with permission from Elsevier.

Molybdenum disulfide (MoS_2_) is possibly the most investigated transition metal dichalcogenide. It is characterised by high surface area, strong NIR absorbance, and thickness-dependent band gap, all valuable for bioimaging, drug delivery, and PTT applications [50,51,52]. In the past decade, researchers have been developing various multifunctional magnetic nanoplatforms based on MoS_2_ for multimodal cancer therapy [53]. For instance, Abareshi and Salehi [50] studied the effect of Fe_3_O_4_ nanoparticles on certain MoS_2_ nanoflake properties. Successful incorporation of Fe_3_O_4_ NPs between the MoS_2_ nanoflakes resulted in a magnetic MoS_2_-Fe_3_O_4_ nanocomposite with a saturation magnetisation (Ms) value of 22.38 emu g^−1^, enabling its separation from aqueous solutions. The main application of this nanocomposite was improving photothermal heat generation, thus targeting tumours by applying external heat. Under 808 nm NIR laser irradiation, the nanocomposite reached 50.9 °C at a concentration of 200 ppm after 10 min, significantly higher than MoS_2_ (33.1 °C) or Fe_3_O_4_ (40.4 °C) alone [50]. Further modifications could improve the stability of such a nanoplatform in physiological solutions, as Li et al. [1] showed by modifying a magnetic MoS_2_ system (mMoS_2_) with liposomes, obtaining a complex for combined photo-chemotherapy. Uniform distribution of phosphorus (from lipids) on the mMoS_2_ surface was confirmed by TEM-mapping, matching the distribution of sulphur, molybdenum, and iron (from mMoS_2_), and showed the uniform distribution of iron oxide nanoparticles on MoS_2_ nanosheet (Figure 4a). The mMoS_2_-lipid exhibited high photothermal conversion efficiency, reaching temperatures around 75 °C (comparable to the non-lipid-modified system), and achieved a doxorubicin (DOX) loading of approximately 108%. In vitro experiments showed successful cellular uptake by human breast cancer cells, MCF-7, with concentration-dependent cytotoxicity (~35% cell viability at 50 μg mL^−1^). Combined photo-chemotherapy (mMoS_2_-Lipid-DOX+NIR) resulted in cell viability of approximately 16% (Figure 4d). Quite promising results were also obtained in vivo, where mMoS_2_-lipid accumulated more effectively at tumour sites compared to unmodified mMoS_2_, resulting in significant tumour inhibition while having minimal toxic side effects, with improved biocompatibility attributed to the lipid surface modification. Additionally, magnetic resonance signal intensity was linearly related to the concentration of magnetic 2D nanomaterial, and in vivo T2-weighted MRI indicated higher accumulation of mMoS_2_-lipid in tumour cells, highlighting its potential as an effective treatment system for breast cancer [1].

The study by Shariati et al. [54] demonstrates another example of MoS_2_ modification with magnetite nanoparticles and gold nanorods, resulting in an MFG nanocomposite. Photothermal experiments were performed for 10 min (808 nm continuous wave laser, 1 W cm^−2^), resulting in a stronger response than unmodified structure. DOX loading was enabled with further modification with PEG, and its release was assessed in phosphate buffer saline (PBS) at pH 5.8 and 7.4, with and without NIR laser irradiation. Under NIR irradiation, approximately 70% of DOX was released from the MFG-PEG sample at pH 5.8, compared to 18% without irradiation at the same pH. At physiological pH, 27% of DOX was released under NIR irradiation, whereas only 13% was released without it [54]. These results indicate both pH- and NIR-dependent DOX release, enabling better control over this potential therapy platform.

Alongside MoS_2_, many complexes of tungsten disulfide (WS_2_) have been synthesised as potential drug delivery agents. One such example is the WS_2_/Au-lipid complex [55], which was evaluated for the dual-responsive release of DOX in combined PTT/chemotherapy due to its responsiveness to both NIR light and pH, and reduced cancer survival rates were observed both in vitro (~30% relative cell viability) and in vivo experiments. Tumour reduction in vivo was observed only with combinational therapy, while all other groups showed little to no change in tumour size in Balb/c mice [55]. Hsiao et al. coated WS_2_ with polypyrrole to electrically stimulate the delivery of 5-fluorouracil (5-FU) [56]. While both studies offer valuable insights without incorporating magnetism, the integration of magnetic properties could improve the effectiveness and control of such systems.

Considering magnetic WS_2_, we begin with a promising approach presented a decade ago by Yang et al. [57]. In this study, WS_2_ nanosheets were functionalised with Fe_3_O_4_ nanoparticles via self-assembly, then encapsulated in a mesoporous silica shell functionalised with PEG. The resulting WS_2_-IO@MS-PEG composite exhibited strong near-infrared light and X-ray absorbance, along with superparamagnetism. Doxorubicin loading was high, with intracellular release triggered by NIR-induced photothermal heating at acidic pH, promoting cancer cell eradication. In vivo combined PTT/chemotherapy with WS_2_-IO@MS-PEG/DOX showed almost complete tumour inhibition, significantly higher compared to monotherapies. Similarly to magnetic MoS_2_ lipid modification, modification of magnetic WS_2_ was also performed [58]. DOX-loading capacity was ~180%, and the platform showed great photothermal performance (reaching 60 °C after 10 min at 200 μg mL^−1^) and dual-responsive drug release, achieving 33% release at pH 5 and NIR irradiation after 4 h. In vitro experiments showed cytotoxicity dependence on the concentration of DOX-loaded platform, and in vivo studies indicated higher accumulation with the lipid-coated structure, similar to the behaviour observed for a lipid-coated MoS_2_-based nanosystem. The complex itself, without the drug, showed practically no cytotoxicity, with a value of relative cell viability being over 90% [58].

In addition to photothermal therapy, photodynamic therapy is another non-invasive phototherapy modality where TMDs can play a significant role. In PDT, visible-light-activated photosensitizers produce reactive oxygen species (ROS), causing oxidative damage and cell death. Wang et al. [59] developed a transition-metal-based magnetic nanomaterial that can potentially combine PTT/PDT/chemotherapy. They solvothermally synthesised hollow molybdenum diselenide nanospheres, introduced Fe_3_O_4_ coating, and subsequently combined these with different amounts of the pluronic F127 (MF-2). The nanocomposite showed increased ROS production and enhanced perfluorocarbon (PFC) loading, leading to a threefold increase in ROS generation, which is desirable for hypoxic tumour environments. Moreover, the narrow band gap (1.25 eV) of MoSe_2_ enhances MF-2’s NIR light absorption, resulting in a photothermal conversion efficiency of 66.2%. This value is significantly higher compared to some materials such as MnO_2_ nanosheets (21.4%) [60], but also MoSe_2_ alone (57.9%) [61] and gold-nanoparticle-modified MoSe_2_ nanosheets (62.2%) [62]. It is also comparable to widely studied gold nanorods [63] and gold nanoparticles, which exhibit efficiencies ranging from 22% to 103%, depending on their size and shape [64]. The presence of Fe_3_O_4_ nanoparticles further improved MF-2’s biodegradation through redox reactions, forming water-soluble Mo(VI) oxide species, and DOX loading also showed promising results. Although this is not a 2D system, its potential as a versatile transition-metal-based theranostic agent for integrated PTT/PDT/chemotherapy applications is recognised [59].

To further emphasise the potential of transition metal dichalcogenides as anticancer agents, we will discuss evaluations using density functional theory (DFT) calculations. One study revealed that complex bilayer MSe_2_ and MS_2_ (M = Mo, W) nanomaterials exhibit strong interactions with the β-lapachone anticancer drug, suggesting their potential for effective drug delivery based on their electronic properties [65]. Certain TMDs can also improve the drug’s effectiveness in treating cancer cells. Specifically, WS_2_ and WSe_2_ interact with proteins either on the cell’s surface or within the cytoplasm, triggering signalling pathways that initiate autophagy. When A549 lung cancer cells were pre-treated with WS_2_ or WSe_2_, they became more susceptible to the effects of doxorubicin, reducing the cancer cells’ resistance to the drug and making the treatment more effective [66]. In vitro studies have shown that TMDs, such as WS_2_ nanosheets, exhibit strong biocompatibility by localising within the cell cytoplasm and being surrounded by membranes rather than inside the nucleus. Cytotoxicity and genotoxicity assessments using human kidney cells showed that WS_2_ did not induce significant levels of ROS or mutations in *S. Typhimurium* bacteria, even at high concentrations and extended exposure times, indicating minimal cytotoxicity and its potential for diverse biomedical applications [67]. Although several studies have reported favourable biocompatibility profiles for TMD-based nanoplatforms, the potential cytotoxic effects, particularly those arising from metal constituents and prolonged exposure, remain an important consideration in their development for clinical applications.

## 3. Transition Metal Carbides/Nitrides

**Figure 5:**
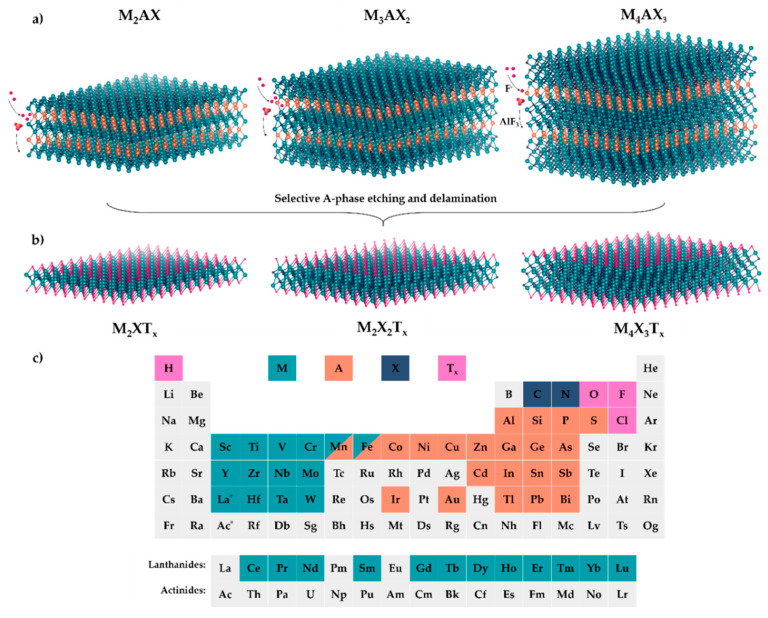
Schematics of three types of MXenes and the top-down approach for their synthesis. (**a**) Structure of MAX phases M_2_AX, M_3_AX_2_, and M_4_AX_3_ and the selective etching of A-layer. (**b**) MXene layers (1-, 2-, or 3-atom thick) obtained after selective etching and their surface termination, T (functional groups). (**c**) Elements that build MAX phases, and T elements in MXenes. Adapted from [73].

**Figure 6:**
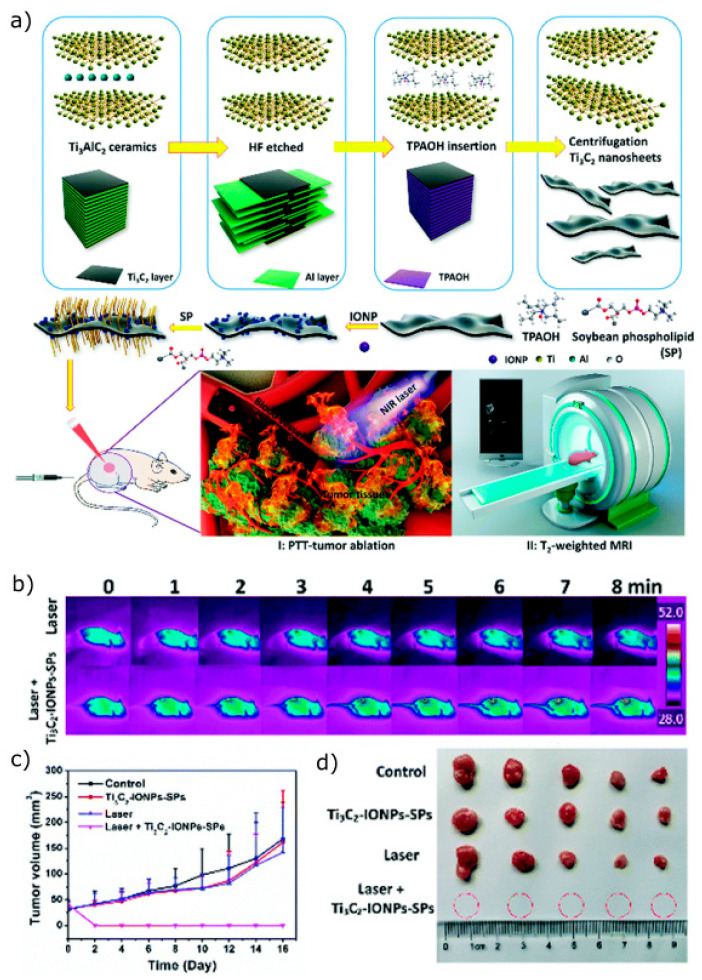
(**a**) Schematics of the exfoliation and surface modification process used to obtain magnetic 2D soybean-modified Ti_3_C_2_ nanocomposite, highlighting its multifunctional capabilities for tumour theranostics, including T2-weighted MRI-guided photothermal therapy. (**b**) Infrared thermal images of 4T1 tumour-bearing mice captured before and after intravenous administration of soybean-phospholipid-modified magnetic nanosheets, further irradiated with 808 nm laser for 8 min (1.5 W cm^−2^). (**c**) Tumour growth curves for different groups of 4T1 tumour-bearing mice subjected to different treatments: control, soybean-phospholipid-modified magnetic Ti_3_C_2_, laser, and laser + soybean-phospholipid-modified magnetic Ti_3_C_2_. (**d**) Representative photographs of excised 4T1 from each treated group after performed photothermal therapy. Reprinted from [91] with permission from RSC Publishing.

Transition metal carbides and nitrides (MXenes) are a novel class of 2D inorganic compounds, structurally similar to graphene sheets. MXenes are typically composed of an early transition metal (e.g., Ti, Mo, V) combined with carbon or nitrogen (X), following three common stoichiometries: M_2_XT_x_, M_3_X_2_T_x_, and M_4_X_3_T_x_ [68] (Figure 5). Their general formula is M_n+1_X_n_T_x_, where T represents surface functional groups (e.g., hydroxyl (-OH), fluorine (-F), and oxygen (-O)), and n typically ranges from 1 to 4 [31]. MXenes, exemplified by Ti_3_C_2_, Mo_2_C, V_2_C, Nb_2_C, Zr_3_C_2_, and Ta_4_C_3_, are commonly produced by selectively etching the A-layer from atomically laminated ceramics known as MAX phases (Figure 6a) [69]. In these MAX phases, “A” refers to elements from groups IIIA (13) to VIA (16) (such as Al, Ga, Si, and Ge) [70,71]. The unique edge-sharing [M₆X] octahedral structure of MAX phases contributes to the stability and properties of the resulting MXenes [72]. More than 70 types of MXenes have been synthesised with various elements, and over 100 types have been theoretically predicted [5].

These 2D materials have attracted wide interest since their discovery in 2011, owing to their physicochemical properties such as high specific surface area, tuneable surface chemistry, electrical conductivity, magnetic properties, low toxicity, luminescence, and high biocompatibility; hence, they have emerged as promising candidates for various bio-applications [74,75]. The presence of surface-terminating functional groups allows the grafting of other molecules and compounds to their surface, providing active sites for drug loading and enabling surface modification and functionalisation [4,71]. These surface functional groups allow active targeting to tumour cells, while passive targeting via the EPR effect supports accumulation in the tumour. MXene-based nanoplatforms impose anticancer effects primarily through combined photothermal and photodynamic therapy, enhanced by their high surface area and tuneable surface chemistry, enabling efficient drug loading and controlled release. That is why MXenes have found applications in photodynamic therapy, photothermal/chemo-photothermal therapy, tissue engineering, regenerative medicine, bioimaging and biosensing, targeted delivery of anticancer drugs [76,77] (including mitigation of drug toxicities), and optimisation of the pharmacokinetics of therapeutic agents [4].

MXenes also have considerable potential to achieve intrinsic magnetism owing to their chemical and structural diversity [78]. The magnetic properties of MXenes are determined by their structure and chemical composition, primarily the occupation of the d-orbitals. Strong covalent M-X and M-T bonds affect the magnetism of MXenes, which typically do not exhibit spontaneous magnetism. However, some pristine MXenes do exhibit magnetic order [79], and the ground state of some MXenes is ferromagnetic, mostly Cr-based ones, such as Cr_2_CF_2_, Cr_2_C(OH)_2_, Cr_2_NF_2_, Cr_2_N(OH)_2_, and Cr_2_NO_2_ [80]. Gao and Zhang [81] investigated the 2D in-plane order of MXenes (i-MXenes) based on DFT calculations and observed that robust magnetism can be achieved by alloying nonmagnetic MXenes with magnetic transition metal elements. Out of the 319 i-MXenes they investigated, about 20% of the compounds exhibit magnetism, with total magnetic moments exceeding 0.2 μB per formula unit in the ferromagnetic configuration, among which 64.5% have ferromagnetic ground states [81]. Zhang et al. [78] used spin-polarised density functional theory calculations to design and investigate 50 double-transition metal MXenes and reported ferromagnetic half-metallicity for some of them.

Through systematic studies performed both in vitro and in vivo, the engineered MXenes and MXene-based nanoplatforms have demonstrated high efficacy in targeted drug delivery and combination therapy in several tumour treatments, with almost all available studies focusing on titanium carbide (Ti_3_C_2_). Li et al. [82] modified the Ti_3_C_2_ surface with a mesoporous silica layer to improve dispersibility, hydrophilicity, controlled drug delivery, and surface chemistry for further potential modification. Systematic studies have revealed that MXene-based nanosystems can actively target tumours through arginine-glycine-aspartic acid (RGD) binding [82,83]. Liu et al. [84] also demonstrated MXenes’ potential to eradicate cancer cells and tumour tissue through combined PTT/PDT/chemotherapy. They synthesised ∼100 nm Ti_3_C_2_ nanosheets with a stable surface functional group Al(OH)^4−^, achieved by supplying additive Al^3+^ to avoid Al loss from long-term etching of Ti_3_AlC_2_ using TMAOH organic base. Layer-by-layer surface modification with hyaluronic acid (HA) and DOX resulted in a multifunctional nanoplatform that could actively target CD44+ overexpressed tumour cells, a characteristic feature of various cancers, associated with tumour progression, metastasis, and resistance to chemotherapy [85]. The overexpression of CD44+ on tumour cells allows HA to enhance the selective delivery of DOX, improving drug uptake in malignant cells while minimising toxicity to healthy tissues. Molecular interactions between DOX and Ti_3_C_2_ nanosheets resulted in 84.2% drug loading capacity. The 178 nm Ti_3_C_2_-DOX complex was able to accumulate at the tumour sites via the EPR effect and ablate the tumour at a low dose (Ti_3_C_2_ at 2 mg kg^−1^ with DOX loaded at 1.6 mg kg^−1^) under a 0.8 W cm^−2^ power of 808 nm NIR laser [84]. Another study explored modification of Ti_3_C_2_ with soybean phospholipid, particularly because it enables easier transport of Ti_3_C_2_ nanosheets within blood vessels, while also keeping them highly dispersed. Drug loading was impressively high, 211.8%, and the platform exhibited both pH-responsive and NIR-laser-triggered on-demand DOX release [86].

The studies presented in this review showed promising theranostic potential; however, control and targeting specificity in cancer treatment remain a challenge [87]. Regardless of their potential to exhibit intrinsic magnetism themselves, none of the experimentally produced MXenes has exhibited strong ferro- or ferrimagnetism. Inducing stronger magnetism can be accomplished by incorporation and growth of Fe_3_O_4_ or ferrites (CuFe_2_O_4_) nanoparticles [88]. Sobolev et al. [31] demonstrated a method for large-scale production of magnetic MXene-based nanocomposites by delaminating multilayer Ti_3_C_2_T_x_ sheets and directly growing iron oxide magnetic nanoparticles within their interlayer spacing. The growth of the Fe_3_O_4_ on the surface of Ti_3_C_2_T_x_ flakes results in higher crystallinity of Fe_3_O_4_ compared to separately synthesised nanoparticles. Higher iron salt concentrations accelerate delamination, creating more nucleation sites and smaller Fe_3_O_4_ particles, while slower delamination leads to fewer nucleation sites, causing the formation of larger crystallites on multilayer MXene structures. This affects magnetic properties, as smaller Fe_3_O_4_ particles on MXene surfaces exhibit higher crystallinity, increasing their saturation magnetisation. Separately synthesised Fe_3_O_4_ NPs show superparamagnetic behaviour with an Ms value of 39 A m^2^ kg^−1^ due to an amorphous phase, whereas Ti_3_C_2_T_x_ enhances Ms to ~45 A m^2^ kg^−1^. A particle mass fraction comparable to or higher than MXenes accelerates delamination, single-layer MXene yield, and magnetic properties [31]. Liu et al. [89] developed a Ti_3_C_2_-CoNWs heteronanocarrier by intercalating cobalt nanowires in Ti_3_C_2_ nanosheets via ultrasound. Ti_3_C_2_-CoNWs exhibited tuneable magnetic properties and high drug loading efficiency of 225.05%, comparable to previously mentioned non-magnetic Ti_3_C_2_ modified with soybean phospholipid, which achieved a DOX loading of 211.8% [86]. Furthermore, drug release from magnetic Ti_3_C_2_-CoNWs was triggered by pH (4–6) and NIR irradiation, inducing simultaneous heat generation and DOX release. Under 808 nm laser irradiation, the combined therapy drastically reduced cancer cell viability to 15%, whereas PTT and chemotherapy alone resulted in around 60% viability each. The heterogeneous system also enhances Ti_3_C_2_ nanosheets’ photothermal performance by improving photoelectron transmission [89].

Magnetic MXenes have also been explored for MR imaging-guided photothermal therapy in cancer treatment. Incorporating Fe_3_O_4_ nanoparticles, either in situ or post-synthesis, enhances their magnetic properties by increasing magnetic saturation and overall magnetic moment, resulting in improved responsiveness to an external magnetic field [90]. Liu et al. [91] synthesised a soybean-phospholipid-modified Ti_3_C_2_-Fe_3_O_4_ composite with a high T2 relaxivity (394.2 mM^−1^ s^−1^), making it a strong candidate for tumour imaging. This magnetic 2D nanocomposite also exhibited a photothermal conversion efficiency of 48.6%, demonstrating effectiveness in vitro and in vivo (4T1 breast cancer xenografts in nude mice) (Figure 6b). Post-treatment observations revealed complete tumour ablation without recurrence, suggesting the potential for multimodal application (Figure 6c,d).

The potential for biomedical applications of MXenes is similar to that of TMDs in terms of their mechanisms of action. Both material groups exhibit strong photothermal conversion efficiency and ROS generation under NIR irradiation or chemical stimuli. While both support drug delivery and combination therapies, MXenes’ tuneable surface chemistry with diverse functional groups further enhances their potential in responsive and multifunctional anticancer platforms [92].

Controlling drug delivery also depends on the coating’s response to stimuli. A hydrogel, combining covalently cross-linked poly(N-isopropyl acrylamide) (PNIPAM), a temperature-responsive polymer, and ionically cross-linked alginate [93] demonstrates how surface modifications impact controlled release. This system enhances MXenes’ biocompatibility and mechanical properties while enabling drug release by shrinking under NIR or alternating magnetic field (AMF) exposure. Grafting PNIPAM allows AMF-triggered drug release in various transition-metal-based 2D systems [7]. At room temperature, drug release can be inhibited, while AMF triggers a change in polymer conformation, enabling release. Additionally, surface nanopore engineering (such as sol-gel chemistry) can further improve drug loading and release [82]. However, despite these advancements, magnetic MXenes still face challenges such as biodegradability, stability, and limited drug-loading capacity [4].

## 4. Transition Metal Oxides

**Figure 7:**
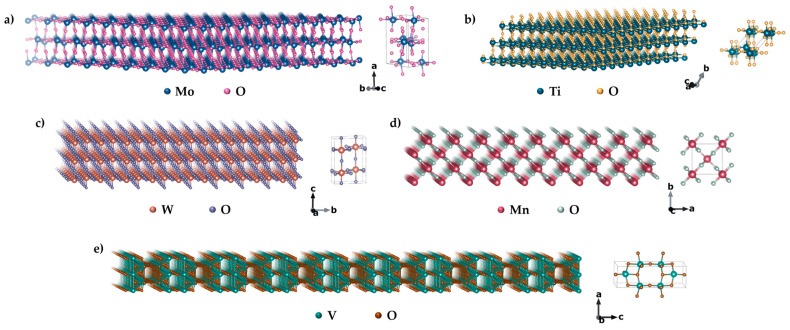
Two-dimensional forms of some transition metal oxides and their unit cells: (**a**) MoO_3_, (**b**) TiO_2_, (**c**) WO_3_, (**d**) MnO_2_, and (**e**) V_2_O_5_.

Transition metal oxides (TMOs) are d-state transition elements oxides with unique magnetic, optical, and electrochemical properties, due to their wide band gaps [27,28,94]. They have atomic-scale to few-atomic-layer thickness, and some can exhibit enhanced magnetism due to quantum confinement and oxygen vacancies (that can also modify electron interactions and surface effects that can modify the spin order) [95,96]. Held together by weak van der Waals forces, these materials can be exfoliated into thin layers, either mechanically or in a liquid phase, which are common top-down approaches for their synthesis. That approach is often followed to obtain materials such as molybdenum trioxide (MoO_3_), manganese dioxide (MnO_2_), and ruthenium oxide (RuO_2_). Bottom-up methods, including self-assembly and chemical vapour deposition, enable controlled design [94]. Besides those mentioned, other unique transition metal oxides include tungsten trioxide (WO_3_), vanadium oxide (VO_2_), titanium dioxide (TiO_2_), and iron oxide forms (FeO) (Figure 7).

The layered structure of these materials allows them to control “light interactions”, leading to photoluminescence and electroluminescence in some of them. The reduced cytotoxicity, along with high surface reactivity and photoelectric properties, makes transition metal oxides promising for combined PTT and PDT [97]. Research on oxygen-deficient forms like MoO_3−x_ and WO_3−x_ has shown that they exhibit localised surface plasmon resonance for improved NIR absorption and high photothermal conversion efficiency, all of which are beneficial for photothermal and photoacoustic imaging. Furthermore, their high atomic numbers enhance X-ray attenuation, making them potential computed tomography (CT) imaging agents [98]. For molybdenum, its trioxide form, MoO_3−x_, stands out for its strong optical absorption in visible and NIR regions, ability to induce caspase-dependent apoptosis, and inhibition of endothelial cell migration [99]. Furthermore, one comparative gene expression study indicated that molybdenum oxide (50–60 nm in size, 1D geometry) showed lower cytotoxicity compared to widely used silver nanoparticles [100], making this material one of the most promising candidates for biomedical applications.

**Figure 8:**
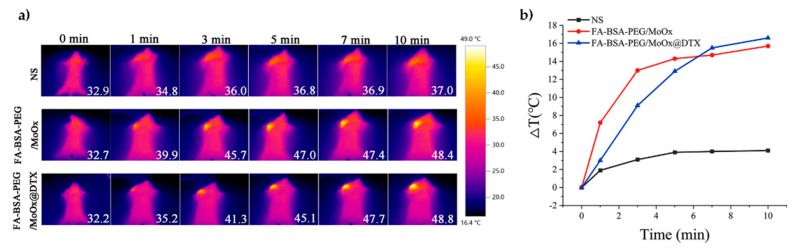
The NIR thermal images of mice (**a**) and corresponding tumour temperature profiles (**b**) showing changes over time for different treatment groups: normal saline, FA-BSA-PEG/MoOx alone, and loaded with docetaxel, respectively. Reprinted from [102]. Copyright Journal of Nanobiotechnology.

Pandey et al. [101] investigated the PTT of solid tumours using bluish-green molybdenum oxide (BG α-MoO_3_), exfoliated from molybdenum oxide powder. Oxygen vacancies were introduced by Xe lamp irradiation to produce blue (B) and then, from them, green (G) nanoflakes. The materials were then functionalised with polypyrrole and irradiated with 808 nm, resulting in temperature increases of 50 °C (BG), 65 °C (B), and 52 °C (G), with corresponding photothermal transduction efficiencies of 29.32%, 44.42%, and 42.00%, respectively [101]. In vitro and in vivo studies showed that all produced materials possess good biocompatibility and photostability, reducing tumour size after 7-day treatment in tumour-bearing mice models. Further optimisation of the stability and solubility of MoO_x_ nanosheets is crucial for their biomedical application. For instance, α-lipoic-acid-conjugated mPEG-NH_2_ and folic-acid-modified bovine serum albumin improve stability and prevent aggregation. Modified blue 2D MoO_3_ achieved 76.49% docetaxel (DTX) loading, inducing immunogenic cell death and inhibiting both primary tumour growth and lung metastasis of breast cancer with an inhibition rate of 93.6%, outperforming Taxotere^®^ alone with fewer side effects [102]. Furthermore, after in vivo NIR irradiation, the tumour temperatures reached 48.4 °C for modified nanocomposite without the drug and 48.8 °C with the drug (Figure 8).

**Figure 9:**
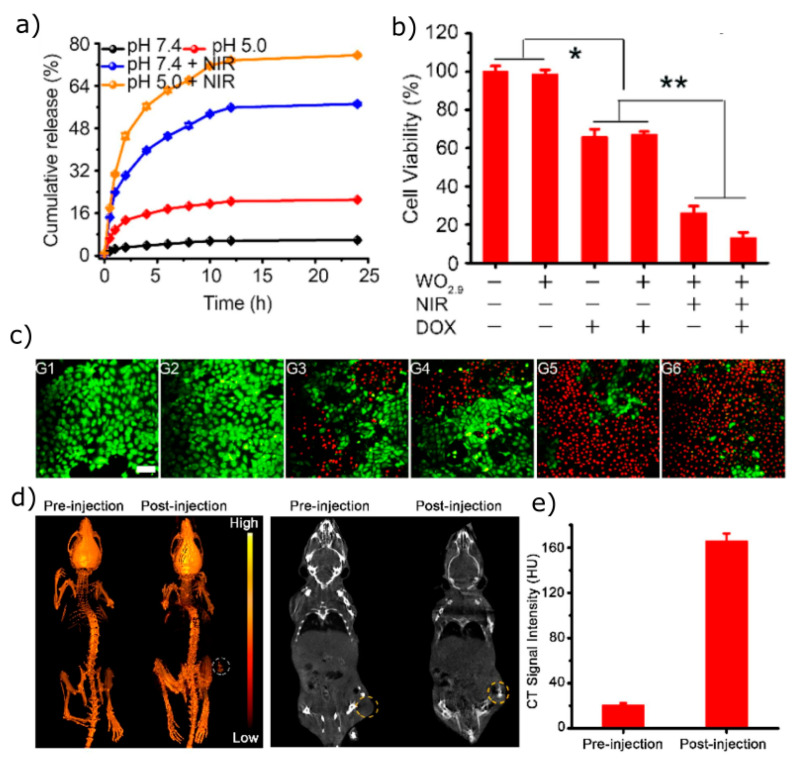
(**a**) Doxorubicin release profiles at different pH conditions with(out) near-infrared-II (1064 nm) laser irradiation. (**b**) Viability of 4T1 cells following various treatments. (* *p* < 0.05, ** *p* < 0.01). (**c**) Fluorescence staining of 4T1 cells (live: green, dead: red) following multiple treatments: G1, control; G2, PEG@WO_2.9_ nanosheets; G3, doxorubicin; G4, PEG@WO_2.9_@DOX nanosheets; G5, PEG@WO_2.9_ nanosheets + NIR; G6, PEG@WO_2.9_@DOX nanosheets + NIR (scale bar = 25 µm). (**d**) Computed tomography imaging of the mice tumours at pre- and post-injection of PEG@WO_2.9_ nanosheets. (**e**) Quantitative analysis of computed tomography signal intensity corresponding to (**d**)). Reprinted from [105] with permission from Elsevier.

Tungsten oxide (WO_3_), another significant TMO, is a promising material for PTT due to the high X-ray absorption coefficient of tungsten (4.438 cm^2^ kg^−1^ at 100 keV) [103]. Researchers have successfully synthesised W_18_O_49_ nanosheets and nanorods whose surface properties could be directed towards catalysis and sensing, and thus biomedical applications. A W_18_O_49_-poly(ε-caprolactone)–poly(ethylene glycol) nanoparticles system with tirapazamine (TPZ) can react with absorbed oxygen to generate ROS when exposed to an 808 nm laser [104]. It also creates a hypoxic tumour microenvironment, activating TPZ for hypoxia-activated chemotherapy, which can be monitored through intracellular ROS detection and in vivo positron emission tomography (PET) imaging. In vivo results showed that this system effectively eliminated solid tumours [104]. Another multimodal PEG-modified tungsten oxide platform, PEGylated WO_2.9_, was developed by Zhang et al. [105] for combined NIR-II-mediated PTT and chemotherapy, achieving a DOX loading efficiency of 102%. Drug release was 3.6-fold higher at pH 5.0 (than at pH 7.4) (Figure 9a), likely due to increased hydrophilicity under acidic conditions. In vitro studies further demonstrated significant cytotoxicity against 4T1 cells, with cell viability reduced to around 16% in the PEG@WO_2.9_@DOX + NIR group (Figure 9b). Fluorescent staining further confirmed these results, showing increased cell death under combined treatment (Figure 9c). Additionally, computed tomography imaging showed enhanced tumour contrast after injection of PEG@WO_2.9_ nanosheets, with quantitative analysis confirming increased CT signal intensity (Figure 9d,e), highlighting their potential as multifunctional agents for cancer theranostics [105].

So far, we have seen that oxygen deficiencies make TMOs sensitive to oxidation, thus making them ideal candidates for PTT, but improvement of their stability in physiological environments, as well as biocompatibility, is required. A PVP-coated W_18_O_49_ system is shown as more biocompatible while maintaining high photothermal conversion efficiency, supporting controlled DOX release (69.1%) under pH and NIR stimuli, with HeLa cell viability dropping to 21.5% [106]. Oxygen vacancies in TMOs also enhance ROS generation, significant for both photodynamic and sonodynamic therapy (SDT), as demonstrated in titanium dioxide (TiO_2_)-loaded black phosphorus nanosheets [107].

Among the various biomedical applications employing magnetism and thermal effects, such as MRI, hyperthermia, and PTT, iron oxide stands out as one of the most significant materials. As an FDA-approved nanomedicine, it has been extensively studied in its 0D form, not only as a contrast agent but also for biosensing and immunoassay applications [108,109]. When integrated with other materials, iron oxide nanoparticles enhance multimodal treatments, making this TMO central to advancements in targeted drug delivery and theranostic platforms. While this review focuses on magnetic 2D TM-based nanomaterials, it is important to acknowledge the potential of 2D iron oxide, particularly in the context of synthesis and biomedical applications. The quantum confinement effects in hematite (α-Fe_2_O_3_) films is between 3 and 12 nm [110], so if the thickness does not exceed these values, it is possible to obtain 2D hematene. Although most studies on 2D Fe-oxides have focused on energy and engineering applications [111], hydrothermal synthesis has been identified as a promising approach for obtaining such films for potential biomedical applications as well [112]. Given iron oxide’s well-established role in magnetic nanomedicine, exploring its 2D geometry could open new possibilities for magnetism-related biomedical applications. This is something that should be further explored, particularly as iron oxide in other-dimensional forms can be integrated with 2D nanomaterials, often outperforming single components [113].

While hydrothermal synthesis is promising for obtaining iron oxides, chemical vapour deposition and liquid exfoliation are more commonly used for the synthesis of 2D nanomaterials in general [114]. Other synthesis techniques such as redox and thermal decomposition have been successful ways of obtaining many TMOs, such as manganese dioxide (MnO_2_) [115]. MnO_2_ ultrathin nanosheets can rapidly increase the temperature, reaching 74.5 °C [116], making it significant for potential PTT applications, also due to the strong NIR adsorption, paramagnetic properties, and reactivity with glutathione (GSH) [117], overall creating opportunities for bioapplications [118]. For instance, Sun et al. [119] recently reviewed 2D MnO_2_ nanosheets’ (~1.2 nm) photothermal conversion efficiency, which was 62.4% under NIR laser irradiation, outperforming multilayer nanosheets (60 nm), which reached only 16.5%. Furthermore, in vitro MRI measurements showed higher values of r1 relaxivity of Mn_3_O_4_ nanoplates compared to 0D nanospheres (2.06 vs. 1.31 mM^−1^ s^−1^, respectively), suggesting advantages of the high surface-to-volume ratio 2D geometry results in [120]. Another TMO that has potential as a tumour therapeutic and diagnostic agent is vanadium oxide (VO_x_) [121]. Selective degradation, resulting in nanoscale products rather than ions, can play a significant role in enhancing excretion and reducing the risk of toxicity [122]. Additionally, vanadium’s multiple valence states could provide controllable redox activity and peroxidase-like activity, which can add up to intracellular oxidative stress [123]. Finally, ROS generation remains a key ability for TMO-based photodynamic therapy, and combining V-based or other TMOs with magnetic nanoparticles could enhance oxidative and thermal stress in cancer cells.

## 5. Metal–Organic Frameworks

**Figure 10:**
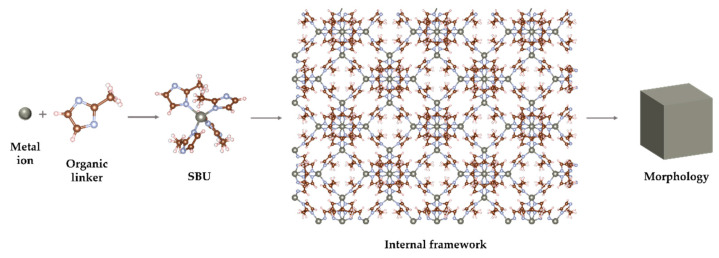
Four hierarchical levels of MOF structure: from metal ions and linkers to secondary building units, the internal framework, and overall topology/morphology.

Metal–organic frameworks (MOFs) are porous coordination polymers—crystalline materials composed of metal ions or clusters (nodes) coordinately bonded by organic ligands (linkers). These materials are particularly interesting due to their structural diversity and multifunctionality [124,125]. Nodes function as connection points, while linkers serve as cross-overs between them, resulting in 1-, 2-, or 3-dimensional networks [126]. The structure of MOFs can be described at four distinct levels (Figure 10). The first level consists of metal ions and linkers. When these basic elements combine, they form secondary building units (SBUs) with geometries such as octahedral or tetrahedral. The tertiary structure refers to the internal framework formed by linking SBUs, while the final level is represented by the overall topology/morphology (size, shape, and orientation), which depends on the growth of the internal framework [125,127].

This architecture results in an impressively high Brunauer–Emmett–Teller (BET) specific surface area, ranging from 3000 to 6000 m^2^g^−1^, with some reports even reporting up to 8000 m^2^g^−1^, making MOFs ideal for high drug-loading capacities [124,128]. Zn, Fe, and Zr are the most commonly employed metals due to their biocompatibility [128], while organic units can range from mono- to tetravalent ligands [125]. The ability to adjust pore characteristics, including size, volume, and surface chemistry, is crucial for biomedical applications [124,129].

Research on MOFs as potential biomedical agents began approximately two decades ago, and although it has predominantly focused on three-dimensional structures, interest in their two-dimensional counterparts is rapidly increasing. Unlike 3D MOFs, 2D MOFs possess a layered structure that offers not only high surface area but also enhanced hydrophilicity and greater interaction with biological agents. These properties make 2D MOFs exceptionally promising candidates for drug delivery and other therapeutic applications. Moreover, their structural features may allow them to outperform 3D MOFs in specific biomedical settings, especially where molecular diffusion and membrane interaction are critical. At the same time, extensive studies on MOFs in general have demonstrated their strong potential for biomedical applications, particularly for cancer drug delivery. Early MOFs had certain limitations regarding the types of drugs they could effectively load due to the lack of larger pores [35]. However, advancements over the years have enabled the design of highly porous MOFs capable of loading high amounts of drugs and supporting controlled drug release by responding to specific stimuli (pH, ATP, UV light) [130,131], allowing them to function as dynamic drug delivery systems. Functionalisation with specific groups further enhances their biocompatibility, controls release kinetics, and reduces toxicity [132,133,134].

Magnetic properties in MOFs can arise from paramagnetic metal centres, particularly V, Cr, Mn, Fe, Co, Ni, and Cu (first-row transition metals). However, combining MOFs with magnetic nanoparticles results in a stronger magnetic response; thus, these highly porous materials are capable of controlled drug release under an external magnetic field, enabling magnetic hyperthermia and magnetic resonance imaging applications [135,136]. For example, core-shell Fe_3_O_4_@HKUST-1(Cu) nanostructure prepared by Ke et al. [137] demonstrated a 16 wt% drug loading capacity for nimesulide, a pancreatic cancer drug, with 0.2 g of drug per 1 g of composite, which was completely released over 11 days at body temperature [137]. Oxaliplatin delivery was also tested using a copper-based MOF combined with Fe_3_O_4_ NPs and showed controlled release, with ~35% oxaliplatin released at pH 1.2 within 30 min [138].

**Figure 11:**
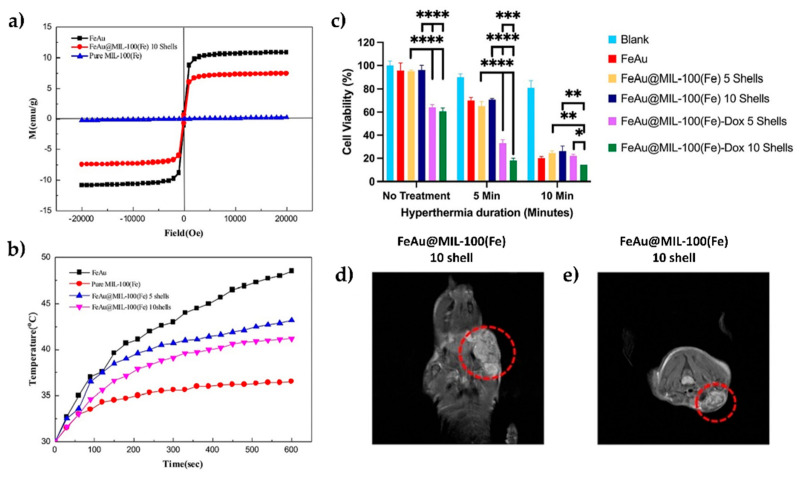
(**a**) Magnetic characterisation of FeAu nanoparticles alone and coated with MIL-100(Fe): M-H curves show reduced Ms following the coating. (**b**) Assessment of hyperthermia performance of FeAu nanoparticles and MIL-100(Fe)-coated FeAu nanostructures with 5 or 10 shells. (**c**) Effect of hyperthermia treatment on cell viability of HSC-3 oral squamous carcinoma cells with(out) doxorubicin-loaded FeAu@MIL-100(Fe); asterisks are representing statistical significance. (**d**,**e**) MRI images of tumour-bearing mice taken 2 h post-injection with FeAu@MIL-100(Fe) 10-shell nanostructures, with tumour regions indicated by red circles. Reprinted from [140] with permission from Elsevier.

In another study, lanthanide-doped MIL-53(Fe)/Fe_3_O_4_ (with La and Gd) was evaluated for antimicrobial and anticancer properties [139]. Cytotoxicity screening on Hep-G2, MCF-7, and HCT-116 showed strong antitumour effects, particularly against MCF-7 cells (IC_50_ = 5.50 ± 0.13 µg mL^−1^). This is attributed to the coordination of metal ions, which improve biological activity by increasing ligand acidity, promoting hydrogen bonding, DNA binding affinity, and oxidative-stress-mediated cancer cell death [139]. MIL-100(Fe) is among the most studied MOFs in general, and different studies have been performed to understand its potential. In one such study, FeAu-nanoparticle-coated MIL-100(Fe) demonstrated nearly complete DOX release (97.19%) and 90% cancer cell death in HSC-3 oral squamous carcinoma cells after 10 min hyperthermia treatment (Figure 11). Figure 11 further provides insights into the material’s properties and therapeutic potential, including its magnetic properties and hyperthermia ability with different numbers of MIL-100(Fe) shells. In vivo studies showed enhanced imaging contrast, 30-fold tumour volume reduction, and improved survival in a mouse model [140]. In another approach, ZIF-8 was explored for tumour imaging and catalytic therapy, after doping it with Fe/Mn [141].

Zr-based MOFs are also gaining attention for drug delivery. Parsaei and Akhbari [142] synthesised Fe_3_O_4_-COOH@UiO-66-NH_2_ via a layer-by-layer assembly method. Fe_3_O_4_-COOH nanoparticles were synthesised, followed by the self-assembly of UiO-66-NH_2_ shell by alternating 15 min ultrasonication of Zr-cluster precursor and NH_2_-BDC solutions, repeated 20 times. The resulting material achieved 43.1% drug loading of quercetin with pH-dependent release behaviour over 11 days [142]. Cytotoxicity assays showed increased apoptosis in MDA-MB-231 breast cancer cells compared to each distinct component. Additionally, MIL-88B-NH_2_ combined with iron oxide nanoparticles was studied for glioblastoma treatments, enabling dual-drug release (carmustine and mertansine), triggered by AFM, with confirmed efficacy in U251 glioblastoma cells [143].

Within the context of ongoing research, 2D MOFs have emerged as a highly promising new class of materials. In addition to the benefits associated with its layered structure, the surface-exposed metal sites, hydrophilicity, and enhanced interactions with cells and biological agents make them highly promising for drug delivery [144,145]. A recent review by Kumar et al. [146] provides an overview of the biomedical application of 2D MOFs—non-magnetic structures that nonetheless possess significant potential for advancement. The following part will discuss some of these directions.

**Figure 12:**
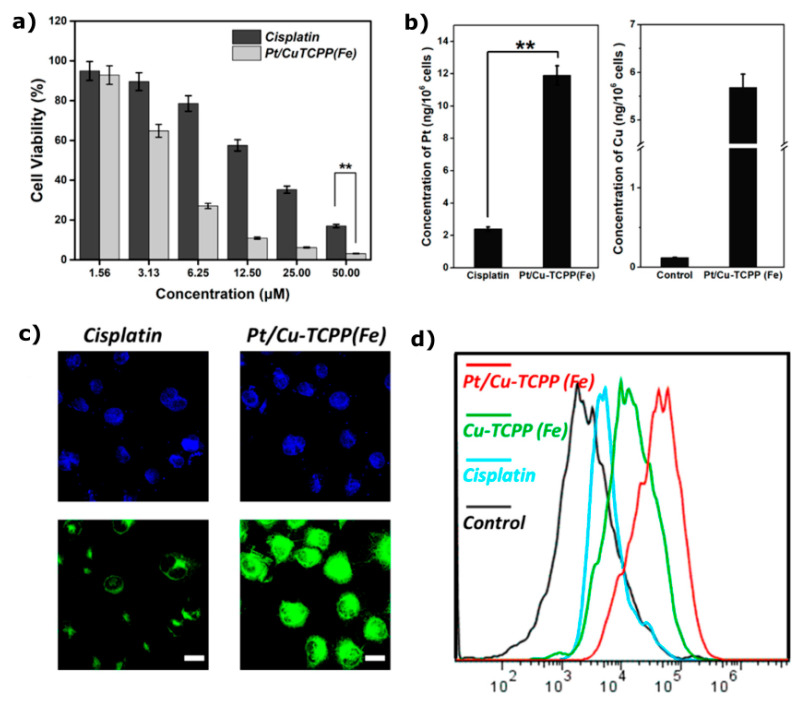
(**a**) Comparison of A549 cell viability after 48 h exposure to cisplatin and Pt/Cu-TCPP(Fe) nanosheets. (**b**) Measurement of intracellular platinum and copper levels following 6 h of incubation with Pt/Cu-TCPP(Fe). (**c**) Assessment of ROS production induced by cisplatin and Pt/Cu-TCPP(Fe) nanosheets; cell nuclei are stained with DAPI (blue), and ROS are visualised by DCF fluorescence (green) (scale bar = 20 μm). (**d**) Flow-cytometry-based quantification of ROS levels in A549 cells, with data presented as mean ± SD (*n* = 3); statistical significance: ** *p* < 0.01. Reprinted with permission from [147]. Copyright 2018 American Chemical Society.

For instance, Li et al. [147] synthesised a 2D iron-porphyrin-based metal–organic framework with Cu nanosheets loaded with cisplatin. Drug release was triggered by pH-dependent degradation of the nanosheets, successfully delivering cisplatin to lung cancer cells. Cellular uptake of Pt/Cu-TCPP(Fe) was significantly higher than that of the drug alone, and cell viability in A549 cells after 48 h at a 50 μM concentration was extremely low, only a few percentage points, demonstrating the effectiveness of the Pt/Cu-TCPP(Fe) nanosheets. The ROS generation capacity of this 2D nanosystem was also higher compared to the individual components, as measured by flow cytometry (Figure 12). Another recent study used 2D ZIF-8 for controlled delivery of siRNA [148], while a similar platform enabled co-delivery of siRNA and cisplatin to ovarian cancer cells [149]. Specifically, Feng et al. [148] developed a multifunctional PDA-ZIF-8 (PSZ) nanoplatform for the delivery of siRNA, combining photothermal and gene therapy, and guided by photoacoustic/near-infrared dual-modality imaging. The PSZ nanocarriers enabled tumour-specific accumulation of siRNA while preventing premature degradation and release. The release of siRNA was triggered by pH, as the ZIF-8 framework degrades under acidic conditions, such as in the tumour microenvironment. The release profile showed only 13% siRNA release at pH 7.4, with significantly higher release at lower pH values: 52% at pH 6.5 and 63% at pH 5.0 after 24 h. This 2D MOF nanosystem exhibited a photothermal conversion efficiency of 39%, with a 30 °C temperature increase at a PSZ concentration of 50 μg mL^−1^, and remained stable over five cycles of photoirradiation. In vivo studies demonstrated complete ablation of tumours in HeLa tumour-bearing mice after combining PSZ+PTT therapy, with no recurrence observed for 10 days [148].

Apart from drug delivery, 2D MOFs are also promising for bioimaging and PDT applications. Zhu et al. [150] developed a 2D Zn-TCPP@PEG nanoplatform for combined chemo-photodynamic therapy. These 2D nanosheets exhibited superior properties compared to their 3D counterparts, including enhanced light-triggered ^1^O_2_ generation for photodynamic therapy, higher drug loading capacity for doxorubicin, increased cellular uptake, and higher ROS generation. Labelling with ^99m^Tc enabled in vivo tracking through single photon emission computed tomography (SPECT). In vivo studies revealed significant tumour growth inhibition after treatment with Zn-TCPP@PEG/DOX nanosheets under light irradiation, demonstrating a successful synergistic anti-tumour effect from combinational photodynamic/chemotherapy. Importantly, the system showed no long-term toxicity, as confirmed by hematoxylin and eosin staining and organ slice examination [150]. Biodegradation studies further indicated that these 2D metal–organic frameworks undergo renal excretion and are not retained long-term in the body, highlighting their biodegradability and potential for clinical applications in cancer therapy.

Despite not being widely explored as magnetic nanosystems, 2D MOFs’ unique properties suggest the potential to create a window of opportunities in the area of bionanomedicine. The layered structure enables efficient interactions with small molecules and biological systems, potentially outperforming materials with three-dimensional geometry in selected applications. Furthermore, 2D MOFs can be hybridised with other 2D materials, such as MXenes or TMDs, to create multifunctional composites with improved stability and drug-loading capacity, and better targeting capabilities [151]. Although challenges remain, such as the scale-up of their synthesis, stability, and long-term effects, overcoming them could enable magnetic 2D MOFs to make significant advancements in cancer therapy applications [20,142].

## 6. Future Directions

While prior reviews have often focused on individual categories of 2D nanomaterials for cancer therapy or broadly addressed their biomedical applications [21,146,152,153,154], this review provides a comparative analysis specifically of 2D nanomaterials containing transition metals in their structure. It also covers their magnetic nanocomposites—an area of growing relevance, as magnetic functionality is increasingly recognised as powerful stimuli for enhancing targeting, enabling controlled drug release, and integrating multimodal therapeutic strategies. By examining four major classes of magnetic 2D nanomaterials based on transition metals—TMDs, MXenes, MOFs, and TMOs—this review highlights both their common advantages and material-specific limitations.

Although the multifunctionality of magnetic 2D transition-metal-based nanomaterials is well established, several critical challenges must be addressed to advance their clinical translation. As inorganic materials, one of the primary concerns is their limited biodegradability and the potential for long-term bioaccumulation, raising toxicity concerns. Bio-inspired surface engineering strategies, such as PEGylation, as demonstrated with PEG-WO_2.9_ nanosheets [105], and modification with lipids, different polymeric coatings, hydrogels, and scaffolds have shown improved biocompatibility and hydrophilicity [155]. Notably, in the case of PEG modification of several TMDs, only MoS_2_ degrades and is excreted from the system within a month due to its unique chemistry [156]. Additionally, agglomeration issues can be addressed by incorporating 2D materials into polymer matrices. For example, TiO_2_@MXene nanosheets can catalyse the polymerisation of acrylic acid monomers and chemically cross-link polymer chains, forming stable hydrogels that enhance dispersion and performance [157].

Moreover, the complexity and heterogeneity of tumours call for more personalised nanoplatforms [158]. The integration of biomolecule-conjugated nanostructures for receptor-targeted delivery or gene-editing tools such as CRISPR/Cas9 systems could enhance the treatment specificity and therapeutic efficacy [159]. Computational approaches, including molecular modelling, have the potential to provide valuable insights into biological interactions and guide the rational design and optimisation of these materials [160].

Nevertheless, the lack of a standardised framework for evaluating long-term in vivo stability and metabolic clearance has delayed their advancement in clinical translation. Systematic toxicological assessments, including organ-specific accumulation profiles and immunotoxicity analyses, are essential to bridging the gap between preclinical efficacy and clinical safety. Furthermore, addressing challenges associated with scalable and reproducible production is critical for enabling broader clinical application. By integrating these strategies with the intrinsic advantages of magnetic 2D transition-metal-based nanomaterials, these platforms hold strong promise for advancing biomedical applications and the broader field of nanotechnology.

## 7. Conclusions

The development of 2D transition-metal-based nanomaterials with intrinsic and extrinsic magnetic properties opens new opportunities for bionanomedicine. Each class of these materials—transition metal dichalcogenides, transition metal carbides/nitrides, metal–organic frameworks, and transition metal oxides—offers distinct advantages for specific biomedical applications. Incorporation of magnetic nanoparticles further enables multimodal therapeutic strategies with enhanced targeting and efficacy, by improving hyperthermia and photothermal treatments. When integrated with 2D materials like transition metal dichalcogenides or carbides/nitrides, they facilitate multimodal therapy, increasing tumour cell death while minimising damage to healthy tissues. MoS_2_ modified with magnetite nanoparticles achieved 70% doxorubicin release under NIR irradiation at pH 5.8 [54], while different lipid-modified magnetic TMDs showed minimal toxic side effects [1,58]. Among the systems discussed in this review, soybean-phospholipid-modified Ti_3_C_2_-Fe_3_O_4_ MXene composite stands out, achieving a photothermal conversion efficiency of 48.6% and a T2 relaxivity of 394.2 mM^−1^s^−1^, resulting in complete tumour ablation [91]. MXenes remain one of the few 2D platforms capable of efficiently integrating photothermal therapy, drug and gene delivery, and MRI imaging within a single system.

As for the drug delivery capabilities, MOFs are exceptionally promising due to their high drug-loading capacity, tuneable porosity, and stimuli responsiveness. Systems such as MIL-100(Fe)-Au achieved 97% DOX release, 90% cancer cell death, and a 30-fold tumour volume reduction [140], matching or outperforming other nanosystems like dendrimer-functionalised nanodiamonds (~95% release) [161], temperature- and pH-responsive liposomes (up to 98% release) [162], and other liposomal-based systems [163,164,165,166]. Furthermore, the increasing interest in 2D MOFs, whose layered structure could improve interaction with biological molecules, is a promising direction, and they could even outperform their 3D counterparts in certain biomedical applications [150]. Multimodality positions both 2D and 3D MOFs as very promising materials for precision oncology, with their potential expected to continue expanding.

Magnetic transition metal oxides and their composites also present strong candidates for multimodal therapy by mainly combining photothermal and photodynamic therapy. Systems like blue MoO_3_ nanosheets achieve photothermal efficiencies of 44.42% and temperatures up to 65 °C [101], outperforming many studied platforms. PVP-modified W_18_O_49_ nanosheets demonstrated responsive DOX release and significant reduction in cancer cell viability [106]. Meanwhile, MnO_2_-based systems generate reactive oxygen species and exhibit photothermal efficiencies over 60% [119], matching or exceeding widely studied photothermal agents like 1D or 0D gold-based nanomaterials and their nanocomposites [167,168]. MnO_2_ and MoO_3_ nanosystems achieved temperatures up to 74.5 °C [116] and 65 °C [101], respectively, surpassing the heat generation capabilities of multiwall carbon nanotube composites [169].

To conclude, each of the four magnetic 2D material classes offers distinct advantages and faces unique limitations in cancer therapy. TMDs are well-suited for photothermal and photodynamic applications due to their strong optical properties, although their functionalisation range can be more limited. MXenes offer superior surface tunability for combinatorial therapy and imaging but may require strategies to address oxidative degradation. Ultra-high drug-loading capacities and stimuli-responsive release is the strongest trait of MOFs, but this group faces challenges with long-term stability and synthesis scalability in 2D forms. TMOs demonstrate superior ROS generation and photothermal properties, yet their potential for multifunctional integration is less developed compared to other classes.

Taken together, magnetic 2D TM-based nanomaterials remain highly competitive, particularly for their capacity to integrate drug delivery, PTT, PDT, hyperthermia, and imaging within a single system. Their advantages lie in higher surface-to-volume ratio and increased cellular internalisation compared to other-dimensional nanomaterials, which significantly improves intracellular delivery efficiency. However, translating these properties into clinical success requires addressing limitations such as material biodegradability, long-term stability, as well as manufacturing scalability. Moving forward, continued interdisciplinary collaboration and innovation are essential to strategically develop and optimise these systems to improve their biocompatibility and overall enhance treatment outcomes, with strong potential to reshape precision nanomedicine for cancer therapy and related biomedical applications.

## Data Availability

No new data were created or analyzed in this study.

## References

[1] Li J., Yang N., Yang M., Lu C., Xie M. (2022). Development of a Magnetic MoS2 System Camouflaged by Lipid for Chemo/Phototherapy of Cancer. Colloids Surf. B Biointerfaces.

[2] Jouybari M.H., Hosseini S., Mahboobnia K., Boloursaz L.A., Moradi M., Irani M. (2019). Simultaneous Controlled Release of 5-FU, DOX and PTX from Chitosan/PLA/5-FU/g-C3N4-DOX/g-C3N4-PTX Triaxial Nanofibers for Breast Cancer Treatment in Vitro. Colloids Surf. B Biointerfaces.

[3] Yan M., Wu S., Wang Y., Liang M., Wang M., Hu W., Yu G., Mao Z., Huang F., Zhou J. (2024). Recent Progress of Supramolecular Chemotherapy Based on Host–Guest Interactions. Adv. Mater..

[4] Li H., Fan R., Zou B., Yan J., Shi Q., Guo G. (2023). Roles of MXenes in Biomedical Applications: Recent Developments and Prospects. J. Nanobiotechnol..

[5] Zhang W.-J., Li S., Vijayan V., Lee J.S., Park S.S., Cui X., Chung I., Lee J., Ahn S., Kim J.R. (2022). ROS- and pH-Responsive Polydopamine Functionalized Ti3C2Tx MXene-Based Nanoparticles as Drug Delivery Nanocarriers with High Antibacterial Activity. Nanomaterials.

[6] Mahmoud A.M., Deambrogi C. (2025). Advancements in Nanotechnology for Targeted and Controlled Drug Delivery in Hematologic Malignancies: Shaping the Future of Targeted Therapeutics. Appl. Biosci..

[7] Ge X., Mohapatra J., Silva E., He G., Gong L., Lyu T., Madhogaria R.P., Zhao X., Cheng Y., Al-Enizi A.M. (2024). Metal–Organic Framework as a New Type of Magnetothermally-Triggered On-Demand Release Carrier. Small.

[8] Boppana S.H., Kutikuppala L.V.S., Sharma S., Madhavrao C., Rangari G., Misra A.K., Kandi V., Mishra S., Singh P.K., Rabaan A.A. (2024). Current Approaches in Smart Nano-Inspired Drug Delivery: A Narrative Review. Health Sci. Rep..

[9] Akbar M.U., Badar M., Zaheer M. (2022). Programmable Drug Release from a Dual-Stimuli Responsive Magnetic Metal–Organic Framework. ACS Omega.

[10] Sun L., Liu H., Ye Y., Lei Y., Islam R., Tan S., Tong R., Miao Y.-B., Cai L. (2023). Smart Nanoparticles for Cancer Therapy. Signal Transduct. Target. Ther..

[11] Dulińska-Litewka J., Łazarczyk A., Hałubiec P., Szafrański O., Karnas K., Karewicz A. (2019). Superparamagnetic Iron Oxide Nanoparticles—Current and Prospective Medical Applications. Materials.

[12] Shen L., Li B., Qiao Y. (2018). Fe3O4 Nanoparticles in Targeted Drug/Gene Delivery Systems. Materials.

[13] Avasthi A., Caro C., Pozo-Torres E., Leal M.P., García-Martín M.L., Puente-Santiago A.R., Rodríguez-Padrón D. (2020). Magnetic Nanoparticles as MRI Contrast Agents. Surface-Modified Nanobiomaterials for Electrochemical and Biomedicine Applications.

[14] Kianfar E. (2021). Magnetic Nanoparticles in Targeted Drug Delivery: A Review. J. Supercond. Nov. Magn..

[15] Van de Walle A., Figuerola A., Espinosa A., Abou-Hassan A., Estrader M., Wilhelm C. (2023). Emergence of Magnetic Nanoparticles in Photothermal and Ferroptotic Therapies. Mater. Horiz..

[16] Cheng H.-L., Guo H.-L., Xie A.-J., Shen Y.-H., Zhu M.-Z. (2021). 4-in-1 Fe3O4/g-C3N4@PPy-DOX Nanocomposites: Magnetic Targeting Guided Trimode Combinatorial Chemotherapy/PDT/PTT for Cancer. J. Inorg. Biochem..

[17] Nikolovski D., Jeremic M., Paunovic J., Vucevic D., Radosavljevic T., Radojević-Škodrić S., Rakocevic R., Nesic D., Pantic I. (2018). Application of Iron Oxide Nanoparticles in Contemporary Experimental Physiology and Cell Biology Research. Rev. Adv. Mater. Sci..

[18] Wang X., Han X., Li C., Chen Z., Huang H., Chen J., Wu C., Fan T., Li T., Huang W. (2021). 2D Materials for Bone Therapy. Adv. Drug Deliv. Rev..

[19] Chang F., Davies G.-L. (2024). From 0D to 2D: Synthesis and Bio-Application of Anisotropic Magnetic Iron Oxide Nanomaterials. Prog. Mater. Sci..

[20] Davis R., Urbanowski R.A., Gaharwar A.K. (2021). 2D Layered Nanomaterials for Therapeutics Delivery. Curr. Opin. Biomed. Eng..

[21] Anju S., Mohanan P.V. (2021). Biomedical Applications of Transition Metal Dichalcogenides (TMDCs). Synth. Met..

[22] Chen L., Dai X., Feng W., Chen Y. (2022). Biomedical Applications of MXenes: From Nanomedicine to Biomaterials. Acc. Mater. Res..

[23] Hefayathullah M., Singh S., Ganesan V., Maduraiveeran G. (2024). Metal-Organic Frameworks for Biomedical Applications: A Review. Adv. Colloid Interface Sci..

[24] Esfanddarani H.M., Panigrahi M. (2024). Phytosynthesis of Transition (Ni, Fe, Co, Cr, and Mn) Metals and Their Oxide Nanoparticles for Biomedical Applications: A Review. J. Mater. Sci..

[25] Saeed M., Uddin W., Saleemi A.S., Hafeez M., Kamil M., Mir I.A., Sunila, Ullah R., Rehman S.U., Ling Z. (2020). Optoelectronic Properties of MoS2-ReS2 and ReS2-MoS2 Heterostructures. Phys. B Condens. Matter.

[26] Murali A., Lokhande G., Deo K.A., Brokesh A., Gaharwar A.K. (2021). Emerging 2D Nanomaterials for Biomedical Applications. Mater. Today.

[27] Garg T., Dabra N., Hundal J.S., Haseeb A.S.M.A. (2023). Ferroelectric Ceramic-Polymer Nanocomposites for Applications in Dielectric Energy Storage Capacitors. Encyclopedia of Materials: Electronics.

[28] Tyagi A., Banerjee S., Cherusseri J., Kar K.K., Kar K.K. (2020). Characteristics of Transition Metal Oxides. Handbook of Nanocomposite Supercapacitor Materials I Characteristics.

[29] Zhao W., Li A., Zhang A., Zheng Y., Liu J. (2018). Recent Advances in Functional-Polymer-Decorated Transition-Metal Nanomaterials for Bioimaging and Cancer Therapy. ChemMedChem.

[30] Nikitin A.A., Ivanova A.V., Semkina A.S., Lazareva P.A., Abakumov M.A. (2022). Magneto-Mechanical Approach in Biomedicine: Benefits, Challenges, and Future Perspectives. Int. J. Mol. Sci..

[31] Sobolev K., Omelyanchik A., Shilov N., Gorshenkov M., Andreev N., Comite A., Slimani S., Peddis D., Ovchenkov Y., Vasiliev A. (2024). Iron Oxide Nanoparticle-Assisted Delamination of Ti3C2Tx MXenes: A New Approach to Produce Magnetic MXene-Based Composites. Nanomaterials.

[32] Hojjati-Najafabadi A., Mansoorianfar M., Liang T., Shahin K., Wen Y., Bahrami A., Karaman C., Zare N., Karimi-Maleh H., Vasseghian Y. (2022). Magnetic-MXene-Based Nanocomposites for Water and Wastewater Treatment: A Review. J. Water Process Eng..

[33] Rethinasabapathy M., Bhaskaran G., Park B., Shin J.-Y., Kim W.-S., Ryu J., Huh Y.S. (2022). Iron Oxide (Fe3O4)-Laden Titanium Carbide (Ti3C2Tx) MXene Stacks for the Efficient Sequestration of Cationic Dyes from Aqueous Solution. Chemosphere.

[34] Xu Z., Long Q., Deng Y., Liao L. (2018). In Situ Synthesis and Catalytic Application of Reduced Graphene Oxide Supported Cobalt Nanowires. Appl. Surf. Sci..

[35] Liu X., Liang T., Zhang R., Ding Q., Wu S., Li C., Lin Y., Ye Y., Zhong Z., Zhou M. (2021). Iron-Based Metal–Organic Frameworks in Drug Delivery and Biomedicine. ACS Appl. Mater. Interfaces.

[36] Ensoylu M., Deliormanlı A.M., Atmaca H. (2021). Tungsten Disulfide Nanoparticle-Containing PCL and PLGA-Coated Bioactive Glass Composite Scaffolds for Bone Tissue Engineering Applications. J. Mater. Sci..

[37] Chen W., Ouyang J., Liu H., Chen M., Zeng K., Sheng J., Liu Z., Han Y., Wang L., Li J. (2017). Black Phosphorus Nanosheet-Based Drug Delivery System for Synergistic Photodynamic/Photothermal/Chemotherapy of Cancer. Adv. Mater. Deerfield Beach Fla.

[38] Roy S., Bermel P. (2018). Electronic and Optical Properties of Ultra-Thin 2D Tungsten Disulfide for Photovoltaic Applications. Sol. Energy Mater. Sol. Cells.

[39] Ratwani C.R., Zhao S., Huang Y., Hadfield M., Kamali A.R., Abdelkader A.M. (2023). Surface Modification of Transition Metal Dichalcogenide Nanosheets for Intrinsically Self-Healing Hydrogels with Enhanced Mechanical Properties. Small.

[40] Qin B., Ma H., Hossain M., Zhong M., Xia Q., Li B., Duan X. (2020). Substrates in the Synthesis of Two-Dimensional Materials via Chemical Vapor Deposition. Chem. Mater..

[41] Yang A., Blancon J.-C., Jiang W., Zhang H., Wong J., Yan E., Lin Y.-R., Crochet J., Kanatzidis M.G., Jariwala D. (2019). Giant Enhancement of Photoluminescence Emission in WS2-Two-Dimensional Perovskite Heterostructures. Nano Lett..

[42] Xu G., Li J., Zhang S., Cai J., Deng X., Wang Y., Pei P. (2024). Two-Dimensional Nano-Biomaterials in Regulating the Tumor Microenvironment for Immunotherapy. Nano TransMed.

[43] Kim J., Cho H., Lim D.-K., Joo M.K., Kim K. (2023). Perspectives for Improving the Tumor Targeting of Nanomedicine via the EPR Effect in Clinical Tumors. Int. J. Mol. Sci..

[44] Er E., Erk N. (2021). An Electrochemical Nanosensor Using a Screen-Printed Electrode Modified with 1T-MoS2/Nafion for Determination of Renin Inhibitor Aliskiren. J. Electrochem. Soc..

[45] Liu W., Lin Z., Tian S., Huang Y., Xue H., Zhu K., Gu C., Yang Y., Li J. (2021). Plasmonic Effect on the Magneto-Optical Property of Monolayer WS2 Studied by Polarized-Raman Spectroscopy. Appl. Sci..

[46] Norden T., Zhao C., Zhang P., Sabirianov R., Petrou A., Zeng H. (2019). Giant Valley Splitting in Monolayer WS2 by Magnetic Proximity Effect. Nat. Commun..

[47] Ge Y., Yang Y., Zhu Y., Yuan M., Sun L., Jiang D., Liu X., Zhang Q., Zhang J., Wang Y. (2024). 2D TiS2-Nanosheet-Coated Concave Gold Arrays with Triple-Coupled Resonances as Sensitive SERS Substrates. Small.

[48] Li B.L., Li R., Zou H.L., Ariga K., Li N.B., Leong D.T. (2020). Engineered Functionalized 2D Nanoarchitectures for Stimuli-Responsive Drug Delivery. Mater. Horiz..

[49] Liu Y., Liu J. (2017). Hybrid Nanomaterials of WS 2 or MoS 2 Nanosheets with Liposomes: Biointerfaces and Multiplexed Drug Delivery. Nanoscale.

[50] Abareshi A., Salehi N. (2022). The Effect of Fe_3_O_4_ Nanoparticles on Structural, Optical, and Thermal Properties MoS_2_ Nanoflakes. J. Mater. Sci. Mater. Electron..

[51] Zhou Z., Li X., Hu T., Xue B., Chen H., Ma L., Liang R., Tan C. (2022). Molybdenum-Based Nanomaterials for Photothermal Cancer Therapy. Adv. NanoBiomed Res..

[52] Wang Z.-Q., Pan Y.-W., Wu J., Qi H.-B., Zhu S., Gu Z.-J. (2024). A Bibliometric Analysis of Molybdenum-Based Nanomaterials in the Biomedical Field. Tungsten.

[53] Liu T., Shi S., Liang C., Shen S., Cheng L., Wang C., Song X., Goel S., Barnhart T.E., Cai W. (2015). Iron Oxide Decorated MoS2 Nanosheets with Double PEGylation for Chelator-Free Radiolabeling and Multimodal Imaging Guided Photothermal Therapy. ACS Nano.

[54] Shariati B., Goodarzi M.T., Jalali A., Salehi N., Mozaffari M. (2023). Gold Nanorods Incorporated into a MoS_2_/Fe_3_O_4_ Nanocomposite for Photothermal Therapy and Drug Delivery. New J. Chem..

[55] Li J., Qi X., Ye P., Yang M., Xie M. (2022). Construction of WS2/Au-Lipid Drug Delivery System for Multiple Combined Therapy of Tumor. J. Drug Deliv. Sci. Technol..

[56] Hsiao P.F., Anbazhagan R., Tsai H.-C., Krishnamoorthi R., Lin S.-J., Lin S.-Y., Lee K.-Y., Kao C.-Y., Chen R.-S., Lai J.-Y. (2020). Fabrication of Electroactive Polypyrrole-Tungsten Disulfide Nanocomposite for Enhanced in Vivo Drug Release in Mice Skin. Mater. Sci. Eng. C.

[57] Yang G., Gong H., Liu T., Sun X., Cheng L., Liu Z. (2015). Two-Dimensional Magnetic WS2@Fe_3_O_4_ Nanocomposite with Mesoporous Silica Coating for Drug Delivery and Imaging-Guided Therapy of Cancer. Biomaterials.

[58] Xie M., Ye P., Zhao R., Yang M. (2023). Magnetic WS2 Nanosheets Functionalized by Biomimetic Lipids with Enhanced Dispersibility for Combined Photothermal and Chemotherapy Therapy. J. Drug Deliv. Sci. Technol..

[59] Wang Y., Zhang F., Lin H., Qu F. (2019). Biodegradable Hollow MoSe_2_/Fe_3_O_4_ Nanospheres as the Photodynamic Therapy-Enhanced Agent for Multimode CT/MR/IR Imaging and Synergistic Antitumor Therapy. ACS Appl. Mater. Interfaces.

[60] Liu Z., Zhang S., Lin H., Zhao M., Yao H., Zhang L., Peng W., Chen Y. (2018). Theranostic 2D Ultrathin MnO_2_ Nanosheets with Fast Responsibility to Endogenous Tumor Microenvironment and Exogenous NIR Irradiation. Biomaterials.

[61] Lei Z., Zhu W., Xu S., Ding J., Wan J., Wu P. (2016). Hydrophilic MoSe_2_ Nanosheets as Effective Photothermal Therapy Agents and Their Application in Smart Devices. ACS Appl. Mater. Interfaces.

[62] Ha C.H., Hur W., Lee S.J., Lee H.B., Kim D.H., Seong G.H. (2025). Targeted Photothermal Cancer Therapy Using Surface-Modified Transition Metal Dichalcogenides. J. Photochem. Photobiol. Chem..

[63] Wang G., Chen X., Li B., Wu L. (2023). Near-Infrared Photothermal Conversion of Polyoxometalate-Modified Gold Nanorods for Plasmon-Enhanced Catalysis. Inorg. Chem. Front..

[64] Kumar P.P.P., Lim D.-K. (2023). Photothermal Effect of Gold Nanoparticles as a Nanomedicine for Diagnosis and Therapeutics. Pharmaceutics.

[65] Mohammed M.H., Hanoon F.H. (2020). Bilayer MSe2 and MS2 (M = Mo, W) as a Novel Drug Delivery System for β-Lapachone Anticancer Drug: Quantum Chemical Study. Comput. Theor. Chem..

[66] Jin W., Yang T., Jia J., Jia J., Zhou X. (2024). Enhanced Sensitivity of A549 Cells to Doxorubicin with WS2 and WSe2 Nanosheets via the Induction of Autophagy. Int. J. Mol. Sci..

[67] Bahri M., Yu D., Zhang C.Y., Chen Z., Yang C., Douadji L., Qin P. (2024). Unleashing the Potential of Tungsten Disulfide: Current Trends in Biosensing and Nanomedicine Applications. Heliyon.

[68] Mei X., Hu T., Wang Y., Weng X., Liang R., Wei M. (2020). Recent Advancements in Two-Dimensional Nanomaterials for Drug Delivery. WIREs Nanomed. Nanobiotechnol..

[69] Zhou A., Liu Y., Li S., Wang X., Ying G., Xia Q., Zhang P. (2021). From Structural Ceramics to 2D Materials with Multi-Applications: A Review on the Development from MAX Phases to MXenes. J. Adv. Ceram..

[70] Mohajer F., Ziarani G.M., Badiei A., Iravani S., Varma R.S. (2022). Advanced MXene-Based Micro- and Nanosystems for Targeted Drug Delivery in Cancer Therapy. Micromachines.

[71] Huang H., Dong C., Feng W., Wang Y., Huang B., Chen Y. (2022). Biomedical Engineering of Two-Dimensional MXenes. Adv. Drug Deliv. Rev..

[72] Huang H., Feng W., Chen Y. (2021). Two-Dimensional Biomaterials: Material Science, Biological Effect and Biomedical Engineering Applications. Chem. Soc. Rev..

[73] Hong W., Wyatt B.C., Nemani S.K., Anasori B. (2020). Double Transition-Metal MXenes: Atomistic Design of Two-Dimensional Carbides and Nitrides. MRS Bull..

[74] Kong F., He X., Liu Q., Qi X., Sun D., Zheng Y., Wang R., Bai Y. (2018). Further Surface Modification by Carbon Coating for in-Situ Growth of Fe3O4 Nanoparticles on MXene Ti3C2 Multilayers for Advanced Li-Ion Storage. Electrochim. Acta.

[75] Iravani S., Varma R.S. (2021). MXenes for Cancer Therapy and Diagnosis: Recent Advances and Current Challenges. ACS Biomater. Sci. Eng..

[76] Iravani S., Varma R.S. (2022). MXenes in Cancer Nanotheranostics. Nanomaterials.

[77] Zhang Y.-Z., El-Demellawi J.K., Jiang Q., Ge G., Liang H., Lee K., Dong X., Alshareef H.N. (2020). MXene Hydrogels: Fundamentals and Applications. Chem. Soc. Rev..

[78] Zhang Y., Cui Z., Sa B., Miao N., Zhou J., Sun Z. (2022). Computational Design of Double Transition Metal MXenes with Intrinsic Magnetic Properties. Nanoscale Horiz..

[79] Li Y., Lai M., Hu M., Zhao S., Liu B., Kai J.-J. (2022). Insights into Electronic and Magnetic Properties of MXenes: From a Fundamental Perspective. Sustain. Mater. Technol..

[80] Khazaei M., Arai M., Sasaki T., Chung C.-Y., Venkataramanan N.S., Estili M., Sakka Y., Kawazoe Y. (2013). Novel Electronic and Magnetic Properties of Two-Dimensional Transition Metal Carbides and Nitrides. Adv. Funct. Mater..

[81] Gao Q., Zhang H. (2020). Magnetic I-MXenes: A New Class of Multifunctional Two-Dimensional Materials. Nanoscale.

[82] Li Z., Zhang H., Han J., Chen Y., Lin H., Yang T. (2018). Surface Nanopore Engineering of 2D MXenes for Targeted and Synergistic Multitherapies of Hepatocellular Carcinoma. Adv. Mater..

[83] Dutta T., Alam P., Mishra S.K. (2025). MXenes and MXene-Based Composites for Biomedical Applications. J. Mater. Chem. B.

[84] Liu G., Zou J., Tang Q., Yang X., Zhang Y., Zhang Q., Huang W., Chen P., Shao J., Dong X. (2017). Surface Modified Ti3C2 MXene Nanosheets for Tumor Targeting Photothermal/Photodynamic/Chemo Synergistic Therapy. ACS Appl. Mater. Interfaces.

[85] Ziranu P., Pretta A., Aimola V., Cau F., Mariani S., D’Agata A.P., Codipietro C., Rizzo D., Dell’Utri V., Sanna G. (2024). CD44: A New Prognostic Marker in Colorectal Cancer?. Cancers.

[86] Han X., Huang J., Lin H., Wang Z., Li P., Chen Y. (2018). 2D Ultrathin MXene-Based Drug-Delivery Nanoplatform for Synergistic Photothermal Ablation and Chemotherapy of Cancer. Adv. Healthc. Mater..

[87] Darroudi M., Elnaz Nazari S., Karimzadeh M., Asgharzadeh F., Khalili-Tanha N., Asghari S.Z., Ranjbari S., Babaei F., Rezayi M., Khazaei M. (2023). Two-Dimensional-Ti_3_C_2_ Magnetic Nanocomposite for Targeted Cancer Chemotherapy. Front. Bioeng. Biotechnol..

[88] Alsafari I.A., Munir S., Zulfiqar S., Saif M.S., Warsi M.F., Shahid M. (2021). Synthesis, Characterization, Photocatalytic and Antibacterial Properties of Copper Ferrite/MXene (CuFe_2_O_4_/Ti_3_C_2_) Nanohybrids. Ceram. Int..

[89] Liu Y., Han Q., Yang W., Gan X., Yang Y., Xie K., Xie L., Deng Y. (2020). Two-Dimensional MXene/Cobalt Nanowire Heterojunction for Controlled Drug Delivery and Chemo-Photothermal Therapy. Mater. Sci. Eng. C.

[90] Rajan A., Chandunika R.K., Raju F., Joshi R., Sahu N.K., Ningthoujam R.S., Tyagi A.K., Ningthoujam R.S. (2022). Synthesis and Processing of Magnetic-Based Nanomaterials for Biomedical Applications. Handbook on Synthesis Strategies for Advanced Materials: Volume-II: Processing and Functionalization of Materials.

[91] Liu Z., Zhao M., Lin H., Dai C., Ren C., Zhang S., Peng W., Chen Y. (2018). 2D Magnetic Titanium Carbide MXene for Cancer Theranostics. J. Mater. Chem. B.

[92] Lee I.-C., Li Y.-C.E., Thomas J.L., Lee M.-H., Lin H.-Y. (2024). Recent Advances Using MXenes in Biomedical Applications. Mater. Horiz..

[93] Yang X., Zhang C., Deng D., Gu Y., Wang H., Zhong Q. (2022). Multiple Stimuli-Responsive MXene-Based Hydrogel as Intelligent Drug Delivery Carriers for Deep Chronic Wound Healing. Small.

[94] Guan M., Wang Q., Zhang X., Bao J., Gong X., Liu Y. (2020). Two-Dimensional Transition Metal Oxide and Hydroxide-Based Hierarchical Architectures for Advanced Supercapacitor Materials. Front. Chem..

[95] Stemmer S., Millis A.J. (2013). Quantum Confinement in Oxide Quantum Wells. MRS Bull..

[96] Zhang X., Rowberg A.J.E., Govindarajan N., He X., Wandelt K., Bussetti G. (2024). Hydrogen Bond Network at the H_2_O/Solid Interface. Encyclopedia of Solid-Liquid Interfaces.

[97] Yin W., Bao T., Zhang X., Gao Q., Yu J., Dong X., Yan L., Gu Z., Zhao Y. (2018). Biodegradable MoOx Nanoparticles with Efficient Near-Infrared Photothermal and Photodynamic Synergetic Cancer Therapy at the Second Biological Window. Nanoscale.

[98] Zhou M., Liu Y., Su Y., Su Q. (2021). Plasmonic Oxygen Defects in MO3− (M = W or Mo) Nanomaterials: Synthesis, Modifications, and Biomedical Applications. Adv. Healthc. Mater..

[99] Xing Y., Cai Y., Cheng J., Xu X. (2020). Applications of Molybdenum Oxide Nanomaterials in the Synergistic Diagnosis and Treatment of Tumor. Appl. Nanosci..

[100] Wahab R., Siddiqui M.A., Ahmad J., Saquib Q., Al-Khedhairy A.A. (2023). A Comparative Cytological Study of Silver and Molybdenum Oxide Nanostructures against Breast Cancer Cells. J. King Saud Univ. Sci..

[101] Pandey S., Sharma K.H., Sharma A.K., Nerthigan Y., Hang D.-R., Wu H.-F. (2018). Comparative Photothermal Performance among Various Sub-Stoichiometric 2D Oxygen-Deficient Molybdenum Oxide Nanoflakes and In Vivo Toxicity. Chem. Eur. J..

[102] Qiu N., Yang X., Zhang Y., Zhang J., Ji J., Zhang Y., Kong X., Xi Y., Liu D., Ye L. (2021). A Molybdenum Oxide-Based Degradable Nanosheet for Combined Chemo-Photothermal Therapy to Improve Tumor Immunosuppression and Suppress Distant Tumors and Lung Metastases. J. Nanobiotechnol..

[103] Zhou Z., Kong B., Yu C., Shi X., Wang M., Liu W., Sun Y., Zhang Y., Yang H., Yang S. (2014). Tungsten Oxide Nanorods: An Efficient Nanoplatform for Tumor CT Imaging and Photothermal Therapy. Sci. Rep..

[104] Zhao P., Ren S., Liu Y., Huang W., Zhang C., He J. (2018). PL–W18O49–TPZ Nanoparticles for Simultaneous Hypoxia-Activated Chemotherapy and Photothermal Therapy. ACS Appl. Mater. Interfaces.

[105] Zhang L., Zhao S., Ouyang J., Deng L., Liu Y.-N. (2022). Oxygen-Deficient Tungsten Oxide Perovskite Nanosheets-Based Photonic Nanomedicine for Cancer Theranostics. Chem. Eng. J..

[106] Zhao Z., Yang S., Yang P., Lin J., Fan J., Zhang B. (2022). Study of Oxygen-Deficient W18O49-Based Drug Delivery System Readily Absorbed through Cellular Internalization Pathways in Tumor-Targeted Chemo-/Photothermal Therapy. Biomater. Adv..

[107] Du W., Chen W., Wang J., Cheng L., Wang J., Zhang H., Song L., Hu Y., Ma X. (2022). Oxygen-Deficient Titanium Dioxide-Loaded Black Phosphorus Nanosheets for Synergistic Photothermal and Sonodynamic Cancer Therapy. Biomater. Adv..

[108] Schneider M.G.M., Martín M.J., Otarola J., Vakarelska E., Simeonov V., Lassalle V., Nedyalkova M. (2022). Biomedical Applications of Iron Oxide Nanoparticles: Current Insights Progress and Perspectives. Pharmaceutics.

[109] Ansari K., Ahmad R., Tanweer M.S., Azam I. (2024). Magnetic Iron Oxide Nanoparticles as a Tool for the Advancement of Biomedical and Environmental Application: A Review. Biomed. Mater. Devices.

[110] Edvinsson T. (2018). Optical Quantum Confinement and Photocatalytic Properties in Two-, One- and Zero-Dimensional Nanostructures. R. Soc. Open Sci..

[111] Kasbe P.S., Yang M., Bosch J., Bu J., DellaCorte C., Xu W. (2024). Two-Dimensional Iron Oxide/Graphene-Based Nanocomposites as High-Performance Solid Lubricants. 2D Mater..

[112] Amrillah T. (2022). All Shapes and Phases of Nanometer-Sized Iron Oxides Made from Natural Sources and Waste Material via Green Synthesis Approach: A Review. Cryst. Growth Des..

[113] Zanganeh S., Hutter G., Spitler R., Lenkov O., Mahmoudi M., Shaw A., Pajarinen J.S., Nejadnik H., Goodman S., Moseley M. (2016). Iron Oxide Nanoparticles Inhibit Tumour Growth by Inducing Pro-Inflammatory Macrophage Polarization in Tumour Tissues. Nat. Nanotechnol..

[114] Dong J., Liu L., Tan C., Xu Q., Zhang J., Qiao Z., Chu D., Liu Y., Zhang Q., Jiang J. (2022). Free-Standing Homochiral 2D Monolayers by Exfoliation of Molecular Crystals. Nature.

[115] Xia H.-Y., Li B.-Y., Zhao Y., Han Y.-H., Wang S.-B., Chen A.-Z., Kankala R.K. (2022). Nanoarchitectured Manganese Dioxide (MnO2)-Based Assemblies for Biomedicine. Coord. Chem. Rev..

[116] Wang L., Guan S., Weng Y., Xu S.-M., Lu H., Meng X., Zhou S. (2019). Highly Efficient Vacancy-Driven Photothermal Therapy Mediated by Ultrathin MnO2 Nanosheets. ACS Appl. Mater. Interfaces.

[117] Fan H., Yan G., Zhao Z., Hu X., Zhang W., Liu H., Fu X., Fu T., Zhang X.-B., Tan W. (2016). A Smart Photosensitizer–Manganese Dioxide Nanosystem for Enhanced Photodynamic Therapy by Reducing Glutathione Levels in Cancer Cells. Angew. Chem. Int. Ed..

[118] Wu M., Hou P., Dong L., Cai L., Chen Z., Zhao M., Li J. (2019). Manganese Dioxide Nanosheets: From Preparation to Biomedical Applications. Int. J. Nanomed..

[119] Sun Y., Wang Y., Liu Y., Wang H., Yang C., Liu X., Wang F. (2023). Integration of Manganese Dioxide-Based Nanomaterials for Biomedical Applications. Adv. NanoBiomed Res..

[120] Huang C.-C., Khu N.-H., Yeh C.-S. (2010). The Characteristics of Sub 10°Nm Manganese Oxide *T*1 Contrast Agents of Different Nanostructured Morphologies. Biomaterials.

[121] Hu D., Li D., Liu X., Zhou Z., Tang J., Shen Y. (2020). Vanadium-Based Nanomaterials for Cancer Diagnosis and Treatment. Biomed. Mater..

[122] Zhang Z., Guo Z., Ruan Z., Ge M., Cao S., Yuan J., Xu Z., Fan L., Zong M., Lin H. (2024). Two-Dimensional Ultrathin Vanadium Oxide Nanosheets as Catalytic Bactericide. Sci. China Mater..

[123] Zhao R., Zhu Y., Feng L., Liu B., Hu Y., Zhu H., Zhao Z., Ding H., Gai S., Yang P. (2024). Architecture of Vanadium-Based MXene Dysregulating Tumor Redox Homeostasis for Amplified Nanozyme Catalytic/Photothermal Therapy. Adv. Mater..

[124] Osterrieth J.W.M., Fairen-Jimenez D. (2021). Metal–Organic Framework Composites for Theragnostics and Drug Delivery Applications. Biotechnol. J..

[125] Lawson H.D., Walton S.P., Chan C. (2021). Metal–Organic Frameworks for Drug Delivery: A Design Perspective. ACS Appl. Mater. Interfaces.

[126] Raptopoulou C.P. (2021). Metal-Organic Frameworks: Synthetic Methods and Potential Applications. Materials.

[127] Mittal A., Roy I., Gandhi S., Mittal A., Roy I., Gandhi S. (2022). Drug Delivery Applications of Metal-Organic Frameworks (MOFs). Drug Carriers.

[128] Saeb M.R., Rabiee N., Mozafari M., Mostafavi E. (2021). Metal-Organic Frameworks (MOFs)-Based Nanomaterials for Drug Delivery. Materials.

[129] Pham H., Ramos K., Sua A., Acuna J., Slowinska K., Nguyen T., Bui A., Weber M.D.R., Tian F. (2020). Tuning Crystal Structures of Iron-Based Metal–Organic Frameworks for Drug Delivery Applications. ACS Omega.

[130] Lei B., Wang M., Jiang Z., Qi W., Su R., He Z. (2018). Constructing Redox-Responsive Metal–Organic Framework Nanocarriers for Anticancer Drug Delivery. ACS Appl. Mater. Interfaces.

[131] Yang X., Tang Q., Jiang Y., Zhang M., Wang M., Mao L. (2019). Nanoscale ATP-Responsive Zeolitic Imidazole Framework-90 as a General Platform for Cytosolic Protein Delivery and Genome Editing. J. Am. Chem. Soc..

[132] Chen W.-H., Karmi O., Willner B., Nechushtai R., Willner I. (2019). Thrombin Aptamer-Modified Metal–Organic Framework Nanoparticles: Functional Nanostructures for Sensing Thrombin and the Triggered Controlled Release of Anti-Blood Clotting Drugs. Sensors.

[133] Chen W.-H., Luo G.-F., Sohn Y.S., Nechushtai R., Willner I. (2019). Enzyme-Driven Release of Loads from Nucleic Acid–Capped Metal–Organic Framework Nanoparticles. Adv. Funct. Mater..

[134] Liu H., Cai G., Yuan S., Zhou X., Gui R., Huang R. (2024). Platelet Membrane-Camouflaged Silver Metal–Organic Framework Biomimetic Nanoparticles for the Treatment of Triple-Negative Breast Cancer. Mol. Pharm..

[135] Cui R., Zhao P., Yan Y., Bao G., Damirin A., Liu Z. (2021). Outstanding Drug-Loading/Release Capacity of Hollow Fe-Metal–Organic Framework-Based Microcapsules: A Potential Multifunctional Drug-Delivery Platform. Inorg. Chem..

[136] Wen T., Quan G., Niu B., Zhou Y., Zhao Y., Lu C., Pan X., Wu C. (2021). Versatile Nanoscale Metal–Organic Frameworks (nMOFs): An Emerging 3D Nanoplatform for Drug Delivery and Therapeutic Applications. Small.

[137] Ke F., Yuan Y.-P., Qiu L.-G., Shen Y.-H., Xie A.-J., Zhu J.-F., Tian X.-Y., Zhang L.-D. (2011). Facile Fabrication of Magnetic Metal–Organic Framework Nanocomposites for Potential Targeted Drug Delivery. J. Mater. Chem..

[138] Nejad A.K.H., Panahi H.A., Keshmirizadeh E., Fard N.T. (2023). Fabrication of a pH-Responsive Drug Delivery System Based on the Super-Paramagnetic Metal-Organic Framework for Targeted Delivery of Oxaliplatin. Int. J. Polym. Mater. Polym. Biomater..

[139] Abu-Dief A.M., Alrashedee F.M.M., Emran K.M., Al-Abdulkarim H.A. (2022). Development of Some Magnetic Metal–Organic Framework Nano Composites for Pharmaceutical Applications. Inorg. Chem. Commun..

[140] Dhawan U., Tseng C.-L., Wu P.-H., Liao M.-Y., Wang H.-Y., Wu K.C.-W., Chung R.-J. (2023). Theranostic Doxorubicin Encapsulated FeAu Alloy@metal-Organic Framework Nanostructures Enable Magnetic Hyperthermia and Medical Imaging in Oral Carcinoma. Nanomed. Nanotechnol. Biol. Med..

[141] Li C., Ye J., Yang X., Liu S., Zhang Z., Wang J., Zhang K., Xu J., Fu Y., Yang P. (2022). Fe/Mn Bimetal-Doped ZIF-8-Coated Luminescent Nanoparticles with Up/Downconversion Dual-Mode Emission for Tumor Self-Enhanced NIR-II Imaging and Catalytic Therapy. ACS Nano.

[142] Parsaei M., Akhbari K. (2023). Magnetic UiO-66-NH2 Core–Shell Nanohybrid as a Promising Carrier for Quercetin Targeted Delivery toward Human Breast Cancer Cells. ACS Omega.

[143] Attia M., Glickman R.D., Romero G., Chen B., Brenner A.J., Ye J.Y. (2022). Optimized Metal-Organic-Framework Based Magnetic Nanocomposites for Efficient Drug Delivery and Controlled Release. J. Drug Deliv. Sci. Technol..

[144] Chakraborty G., Park I.-H., Medishetty R., Vittal J.J. (2021). Two-Dimensional Metal-Organic Framework Materials: Synthesis, Structures, Properties and Applications. Chem. Rev..

[145] Li K., Ji Q., Liang H., Hua Z., Hang X., Zeng L., Han H. (2023). Biomedical Application of 2D Nanomaterials in Neuroscience. J. Nanobiotechnol..

[146] Kumar S.A., Balasubramaniam B., Bhunia S., Jaiswal M.K., Verma K., Prateek, Khademhosseini A., Gupta R.K., Gaharwar A.K. (2021). Two-Dimensional Metal Organic Frameworks for Biomedical Applications. WIREs Nanomed. Nanobiotechnol..

[147] Li Y., Gao Z., Chen F., You C., Wu H., Sun K., An P., Cheng K., Sun C., Zhu X. (2018). Decoration of Cisplatin on 2D Metal–Organic Frameworks for Enhanced Anticancer Effects through Highly Increased Reactive Oxygen Species Generation. ACS Appl. Mater. Interfaces.

[148] Feng J., Yu W., Xu Z., Hu J., Liu J., Wang F. (2024). Correction to “Multifunctional siRNA-Laden Hybrid Nanoplatform for Noninvasive PA/IR Dual-Modal Imaging-Guided Enhanced Photogenetherapy”. ACS Appl. Mater. Interfaces.

[149] He C., Liu D., Lin W. (2015). Nanomedicine Applications of Hybrid Nanomaterials Built from Metal–Ligand Coordination Bonds: Nanoscale Metal–Organic Frameworks and Nanoscale Coordination Polymers. Chem. Rev..

[150] Zhu W., Yang Y., Jin Q., Chao Y., Tian L., Liu J., Dong Z., Liu Z. (2019). Two-Dimensional Metal-Organic-Framework as a Unique Theranostic Nano-Platform for Nuclear Imaging and Chemo-Photodynamic Cancer Therapy. Nano Res..

[151] Rabiee N., Bagherzadeh M., Jouyandeh M., Zarrintaj P., Saeb M.R., Mozafari M., Shokouhimehr M., Varma R.S. (2021). Natural Polymers Decorated MOF-MXene Nanocarriers for Co-Delivery of Doxorubicin/pCRISPR. ACS Appl. Bio Mater..

[152] Derakhshi M., Daemi S., Shahini P., Habibzadeh A., Mostafavi E., Ashkarran A.A. (2022). Two-Dimensional Nanomaterials beyond Graphene for Biomedical Applications. J. Funct. Biomater..

[153] Huang K., Li Z., Lin J., Han G., Huang P. (2018). Two-Dimensional Transition Metal Carbides and Nitrides (MXenes) for Biomedical Applications. Chem. Soc. Rev..

[154] Liu W., Song X., Jiang Q., Guo W., Liu J., Chu X., Lei Z. (2024). Transition Metal Oxide Nanomaterials: New Weapons to Boost Anti-Tumor Immunity Cycle. Nanomaterials.

[155] Pires L.S., Magalhães F.D., Pinto A.M. (2022). New Polymeric Composites Based on Two-Dimensional Nanomaterials for Biomedical Applications. Polymers.

[156] Martín C., Kostarelos K., Prato M., Bianco A. (2019). Biocompatibility and Biodegradability of 2D Materials: Graphene and Beyond. Chem. Commun..

[157] Wang Q., Pan X., Lin C., Gao H., Cao S., Ni Y., Ma X. (2020). Modified Ti3C2TX (MXene) Nanosheet-Catalyzed Self-Assembled, Anti-Aggregated, Ultra-Stretchable, Conductive Hydrogels for Wearable Bioelectronics. Chem. Eng. J..

[158] Dagogo-Jack I., Shaw A.T. (2018). Tumour Heterogeneity and Resistance to Cancer Therapies. Nat. Rev. Clin. Oncol..

[159] Xu X., Liu C., Wang Y., Koivisto O., Zhou J., Shu Y., Zhang H. (2021). Nanotechnology-Based Delivery of CRISPR/Cas9 for Cancer Treatment. Adv. Drug Deliv. Rev..

[160] Chen S.H., Bell D.R., Luan B. (2022). Understanding Interactions between Biomolecules and Two-Dimensional Nanomaterials Using in Silico Microscopes. Adv. Drug Deliv. Rev..

[161] Patil S., Mishra V.S., Yadav N., Reddy P.C., Lochab B. (2022). Dendrimer-Functionalized Nanodiamonds as Safe and Efficient Drug Carriers for Cancer Therapy: Nucleus Penetrating Nanoparticles. ACS Appl. Bio Mater..

[162] Nezhadali A., Shapouri M.R., Amoli-Diva M. (2020). Anti-Cancer Combination Therapy by Co-Delivery of Hydrophilic and Hydrophobic Using Dual Temperature and pH-Responsive Liposomes. Micro Nano Lett..

[163] Moghimipour E., Rezaei M., Ramezani Z., Kouchak M., Amini M., Angali K.A., Dorkoosh F.A., Handali S. (2018). Transferrin Targeted Liposomal 5-Fluorouracil Induced Apoptosis *via* Mitochondria Signaling Pathway in Cancer Cells. Life Sci..

[164] Wang W.-Y., Cao Y.-X., Zhou X., Wei B. (2019). Delivery of Folic Acid-Modified Liposomal Curcumin for Targeted Cervical Carcinoma Therapy. Drug Des. Devel. Ther..

[165] Hardiansyah A., Huang L.-Y., Yang M.-C., Liu T.-Y., Tsai S.-C., Yang C.-Y., Kuo C.-Y., Chan T.-Y., Zou H.-M., Lian W.-N. (2014). Magnetic Liposomes for Colorectal Cancer Cells Therapy by High-Frequency Magnetic Field Treatment. Nanoscale Res. Lett..

[166] Sonali, Singh R.P., Singh N., Sharma G., Vijayakumar M.R., Koch B., Singh S., Singh U., Dash D., Pandey B.L. (2016). Transferrin Liposomes of Docetaxel for Brain-Targeted Cancer Applications: Formulation and Brain Theranostics. Drug Deliv..

[167] Zhang Y., Zhan X., Xiong J., Peng S., Huang W., Joshi R., Cai Y., Liu Y., Li R., Yuan K. (2018). Temperature-Dependent Cell Death Patterns Induced by Functionalized Gold Nanoparticle Photothermal Therapy in Melanoma Cells. Sci. Rep..

[168] Beik J., Alamzadeh Z., Mirrahimi M., Sarikhani A., Ardakani T.S., Asadi M., Irajirad R., Mirrahimi M., Mahabadi V.P., Eslahi N. (2021). Multifunctional Theranostic Graphene Oxide Nanoflakes as MR Imaging Agents with Enhanced Photothermal and Radiosensitizing Properties. ACS Appl. Bio Mater..

[169] Naief M.F., Khalaf Y.H., Mohammed A.M. (2022). Novel Photothermal Therapy Using Multi-Walled Carbon Nanotubes and Platinum Nanocomposite for Human Prostate Cancer PC3 Cell Line. J. Organomet. Chem..

